# Therapy-induced developmental reprogramming of prostate cancer cells and acquired therapy resistance

**DOI:** 10.18632/oncotarget.14850

**Published:** 2017-01-27

**Authors:** Mannan Nouri, Josselin Caradec, Amy Anne Lubik, Na Li, Brett G. Hollier, Mandeep Takhar, Manuel Altimirano-Dimas, Mengqian Chen, Mani Roshan-Moniri, Miriam Butler, Melanie Lehman, Jennifer Bishop, Sarah Truong, Shih-Chieh Huang, Dawn Cochrane, Michael Cox, Colin Collins, Martin Gleave, Nicholas Erho, Mohamed Alshalafa, Elai Davicioni, Colleen Nelson, Sheryl Gregory-Evans, R. Jeffrey Karnes, Robert B. Jenkins, Eric A. Klein, Ralph Buttyan

**Affiliations:** ^1^ Vancouver Prostate Centre, Vancouver, Canada; ^2^ Department of Urologic Sciences, University of British Columbia, Vancouver, Canada; ^3^ Institute of Health and Biomedical Innovation, Queensland University of Technology, Brisbane, Australia; ^4^ GenomeDX Biosciences, Vancouver, Canada; ^5^ Drug Discovery & Biomedical Sciences, South Carolina College of Pharmacy, Columbia, South Carolina, USA; ^6^ Department of Molecular Oncology, British Columbia Cancer Agency, Vancouver, Canada; ^7^ GenomeDX Biosciences, San Diego, California, USA; ^8^ Department of Ophthalmology and Visual Sciences, University of British Columbia, Vancouver, Canada; ^9^ Department of Urology, Mayo Clinic, Rochester, Minnesota, USA; ^10^ Department of Laboratory Medicine and Pathology, Mayo Clinic, Rochester, Minnesota, USA; ^11^ Glickman Urological and Kidney Institute, Cleveland Clinic Foundation, Cleveland, Ohio, USA

**Keywords:** prostate cancer, cancer stem cell, neural crest, neuroendocrine transdifferentiation, hormone resistance

## Abstract

Treatment-induced neuroendocrine transdifferentiation (NEtD) complicates therapies for metastatic prostate cancer (PCa). Based on evidence that PCa cells can transdifferentiate to other neuroectodermally-derived cell lineages *in vitro*, we proposed that NEtD requires first an intermediary reprogramming to metastable cancer stem-like cells (CSCs) of a neural class and we demonstrate that several different AR^+^/PSA^+^ PCa cell lines were efficiently reprogrammed to, maintained and propagated as CSCs by growth in androgen-free neural/neural crest (N/NC) stem medium. Such reprogrammed cells lost features of prostate differentiation; gained features of N/NC stem cells and tumor-initiating potential; were resistant to androgen signaling inhibition; and acquired an invasive phenotype *in vitro* and *in vivo*. When placed back into serum-containing mediums, reprogrammed cells could be re-differentiated to N-/NC-derived cell lineages or return back to an AR^+^ prostate-like state. Once returned, the AR^+^ cells were resistant to androgen signaling inhibition. Acute androgen deprivation or anti-androgen treatment in serum-containing medium led to the transient appearance of a sub-population of cells with similar characteristics. Finally, a 132 gene signature derived from reprogrammed PCa cell lines distinguished tumors from PCa patients with adverse outcomes. This model may explain neural manifestations of PCa associated with lethal disease. The metastable nature of the reprogrammed stem-like PCa cells suggests that cycles of PCa cell reprogramming followed by re-differentiation may support disease progression and therapeutic resistance. The ability of a gene signature from reprogrammed PCa cells to identify tumors from patients with metastasis or PCa-specific mortality implies that developmental reprogramming is linked to aggressive tumor behaviors.

## INTRODUCTION

Hormone therapies for advanced prostate cancer (PCa) can shrink metastatic lesions, slow tumor growth and significantly extend patient survival. Yet they remain palliative as treated patients usually progress to castration resistant disease (CRPC) that portends lethality [1]. The clinical success of a new generation of anti-androgens in further prolonging survival of CRPC patients demonstrates that promiscuous androgen receptor (AR) activity plays a role in hormone resistance [2]. AR-mediated resistance is complex and can be generated by overexpression/amplification of full-length AR; by mutations in the AR gene or by expression of truncated ARs resulting from altered splicing of AR mRNA [3]. For a subset of patients, resistance can also be generated by a non-AR-based mechanism referred to as neuroendocrine transdifferentation (NEtD) [4, 5]. NEtD is associated with a loss of prostate differentiation, including AR expression, and a gain of neuroendocrine cell features. While neuroendocrine PCa (NEPC) remains a relatively limited condition, its occurrence is increasing under the constraints of stringent anti-androgen therapies and its aggressive nature raises concerns that NEtD will increasingly confound opportunities for disease control.

NEtD is modeled *in vitro* when androgen-dependent PCa cells, particularly LNCaP, are placed into an androgen-deficient growth medium [6, 7]. NEtD is accompanied by an altered cell morphology characterized by small central-nucleated cell bodies with elongated neurite-like extensions and by overexpressions of secretagranins, synaptophysin, neural specific enolase and a variety of neuropeptides [6–8]. NEtD is generally believed to reflect a direct switch of cell states, putatively guided by simple loss(es)/gain(s) of expression or function of neuroendocrine (NE) lineage-regulators such as REST, AURAK or MYCN [8–10]. In this model, the NEtD-inducer regulates the expression of NE lineage determinants that awaken and enforce the NEPC phenotype. Here we present results of an experimental study that contradicts the direct conversion model of NEtD and, instead, argues for a model whereby differentiated PCa cells must first developmentally reprogram back to a neural/neural crest (N/NC) stem cell-like state before re-differentiating to NE-like cells.

Our proposal reflects the growing interest in cancer stem cells (CSCs) as mediators of aggressive prostate tumor behaviors. Putative CSCs have been distinguished and selectively enriched from tumor cell populations based on high expression of certain cell surface antigens (CD49b/29 [integrin-α_2_β_1_], CD133, and CD44) [11, 12]; high expression of detoxifying enzymes [13] or membrane transporters [14] and based upon low expression of the prostate-specific differentiation marker, prostate specific antigen (PSA) [15]. Such cells tend to overexpress Yamanaka Factors Oct3/4 [Pou5f1], Nanog and Sox2 [16]. Enriched populations of CSCs typically have high clonogenicity in culture and the ability to grow in an anchorage-independent manner leading to the formation of multicellular spheroids. While they tend to have slower proliferation rates compared to non-CSCs, they can form tumors from very low numbers of cells xenografted into immune-deficient mice.

CSCs may represent a remnant of the original transforming event within a prostate epithelial stem cell population [17, 18]. Their propagation over time through asymmetric replication, to generate a slower replicating CSC and a more rapid proliferating differentiated cancer cell, would account for the dominance of differentiated cells in tumors [18, 19]. However, there is increasing evidence that CSCs can also be generated through de-differentiation from non-CSCs under the influence of idiopathic activation of polycomb or through activation of an Epithelial to Mesenchymal Transition (EMT) program [19–21]. While evidence supports prostate CSC resistance to androgen deprivation (AD) therapy [11, 15] and anti-mitotics [22], prostate CSCs remain poorly characterized for their ability to metastasize and for other qualities associated with pluripotent/multipotent stem cells such as the ability to differentiate to alternate cell lineages.

Evidence, presented here, of LNCaP cell transdifferentiation to other neural/neural crest (N/NC) stem cell-derived lineages *in vitro*, under chronic AD, caused us to re-evaluate the paradigm of direct NEtD. We detected a complex spectrum of gene expression changes in chronic AD LNCaPs that was consistent with the presence of multiple transdifferentiated N/NC-derived cell lineages rather than exclusively NE-like cells. Indeed, others have already reported that LNCaP cells can acquire a morphological phenotype and transcriptome resembling that of adult neurons [23]. Such a spectrum of transdifferentiation might require an intermediate N/NC stem-like state and we sought to reveal, expand and characterize this intermediary state using an androgen-free N/NC stem cell medium to “reprogram” differentiated PCa cells. By finding the means to efficiently reprogram, without genetic manipulation, maintain and further propagate PCa cells in this novel transitional stem-like state, we have been able to characterize features that show they differ from other types of PCa CSCs and imply that they represent a more aggressive form of PCa.

## RESULTS

### Conditional reprogramming of PCa cells to N/NC stem-like cells with resistance to androgen therapeutics and enhanced invasive/metastatic properties

**Figure 1:**
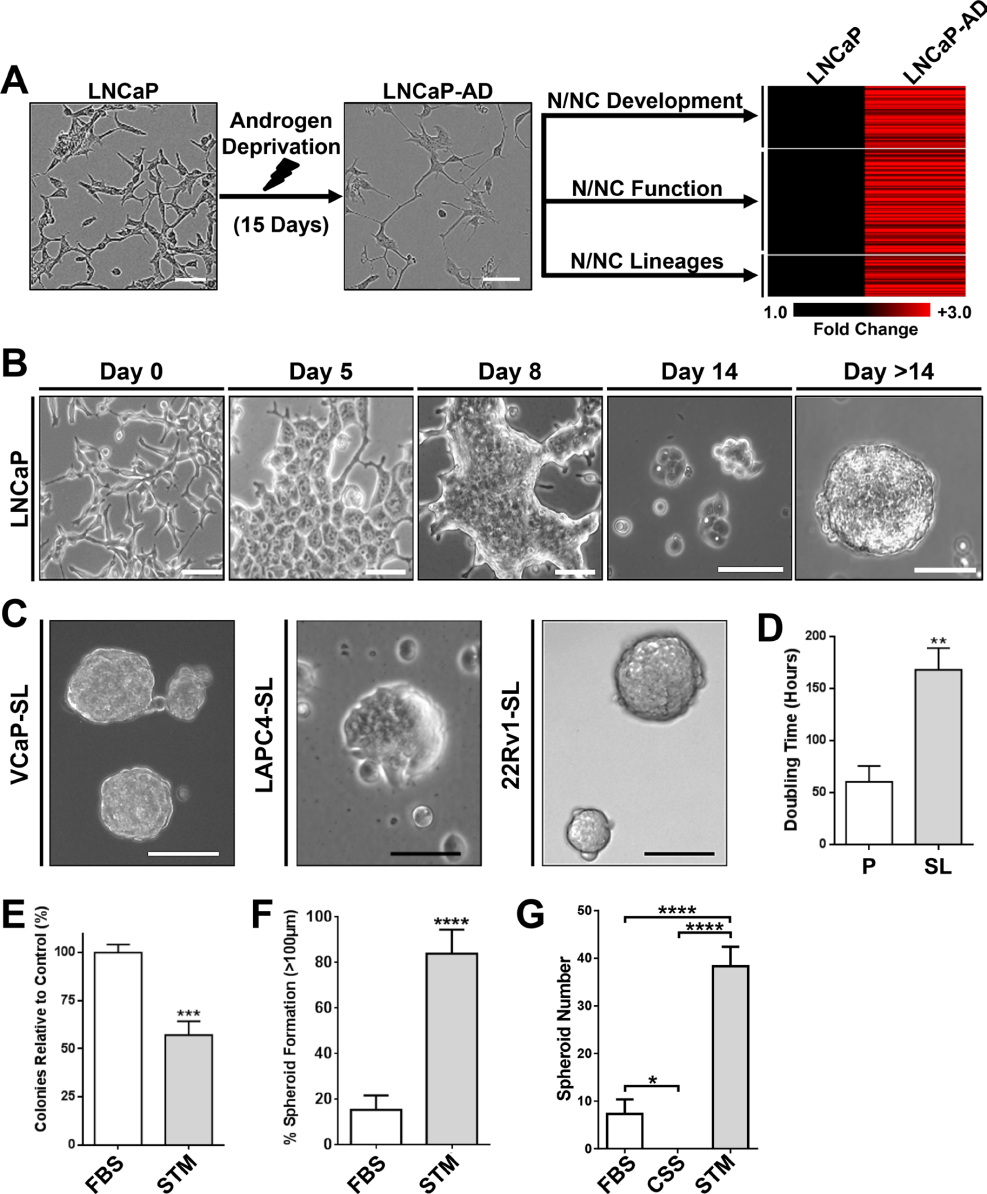
Developmental reprogramming of PCa Cells to a stem-like intermediate (**A**) Chronic AD of LNCaP cells upregulated genes associated with N/NC stem cells and derivative tissues. LNCaP cells (Left) cultured in androgen-depleted medium for 15-days undergo a morphological transformation (Middle), and overexpress genes associated with N/NC stem cells and derivative tissues shown on heat-mapping (Right). (**B**) Representative phase-contrast images of LNCaP cells over two weeks of culture in STM. (**C**) Representative phase-contrast images of VCaP, LAPC4 or 22Rv1 spheroids after 14-day STM-mediated reprogramming. Scale bars represent 100 μm. (**D**) Proliferation assays of parental LNCaP (P) and reprogrammed stem-like LNCaP cells (SL) showed that the doubling time of LNCaP-SL is significantly greater than parentals. Mean ± standard error; **P < 0.01. (**E**) Clone formation of dispersed LNCaP cells grown in FBS or STM medium indicates that reprogramming occurs in at least 57% of LNCaP cells plated. Mean ± standard deviation; ***P < 0.001. (**F**) The percentage of spheroids (diameters greater than 100 μm) is significantly greater in STM cultured LNCaP cells compared to FBS cultured LNCaP cells, over 14 days. Mean ± standard deviation shown; ****P < 0.0001. (**G**) Spheroid colony formation assay of LNCaP cells cultured in FBS, CSS or STM on attachment-free plates shows advantage of STM compared to standard culture medium (RPMI) with (FBS) or without androgen (CSS). Mean ± standard deviation; ***P < 0.001; ****P < 0.0001.

LNCaP cells undergo NEtD under acute AD [6, 7]. We performed comparative gene microarray expression analyses using RNAs from parental or 15-day AD LNCaP cells to identify genes more chronically upregulated after AD. Outcomes showed striking upregulation of genes specific for sensory and peripheral neurons, sensory tissue development and function, facial/tooth development and cardiovascular development amongst others, collectively representing a heterogeneous assortment of potential neuroectodermal cell lineages that arise from N/NC stem cells [24]. Functional annotation of upregulated genes showed that most clustered into three categories that included genes involved in N/NC development, functions of N/NC stem cells and markers of N/NC-derived cell lineages (Figure 1A, Supplementary Table 1). We reasoned that these observations suggested the possibility that transdifferentiation might be mediated by transient reprogramming of PCa cells to an intermediate N/NC stem cell-like state that then re-differentiates into NE and other neuroectodermal-derived cell lineages.

We further reasoned that an androgen-free medium that supports growth of N/NC stem cells might enable conditional reprogramming to this intermediate state and allow us to stably propagate such cells for further analyses. When we cultured any of four AR^+^ PCa cell lines (LNCaP, VCaP, LAPC4 and 22Rv1) in N/NC Stem-Transition Medium (STM), they underwent a marked change in morphology, characterized by transition to cells with a rounded body, enlarged nuclei and strikingly distinct nucleoli (Figure 1B) over the period of a week. Unlike parental PCa cells that tend to separate until confluence, cells placed into STM clustered together over time, forming 3-dimensional mats. Upon transfer into fresh STM, cells thereafter formed small, poorly-attached rosettes that grew into spheroids (Figure 1B, 1C). This non-adherent growth pattern was maintained through further passaging and we have transferred cells as many as 20 times in STM without deviation from this pattern. While cells continue to grow in the absence of androgen, their doubling time is approximately 3 times longer than parentals (Figure 1D). We used colony-forming and sphere-forming assays to quantify the efficiency of this transition for LNCaP cells, indicating a conversion efficiency of approximately 60% and 80% respectively (Figure 1E, 1F). Reprogramming did not occur with standard embryonic stem cell (ESC) medium or when the neural stem supplement was removed (data not shown). When tested in non-adherent plate colony-forming assays, STM also strongly promoted the formation of proliferative spheroidal colonies compared to standard PCa cell culture media, with or without androgen (Figure 1G).

**Figure 2:**
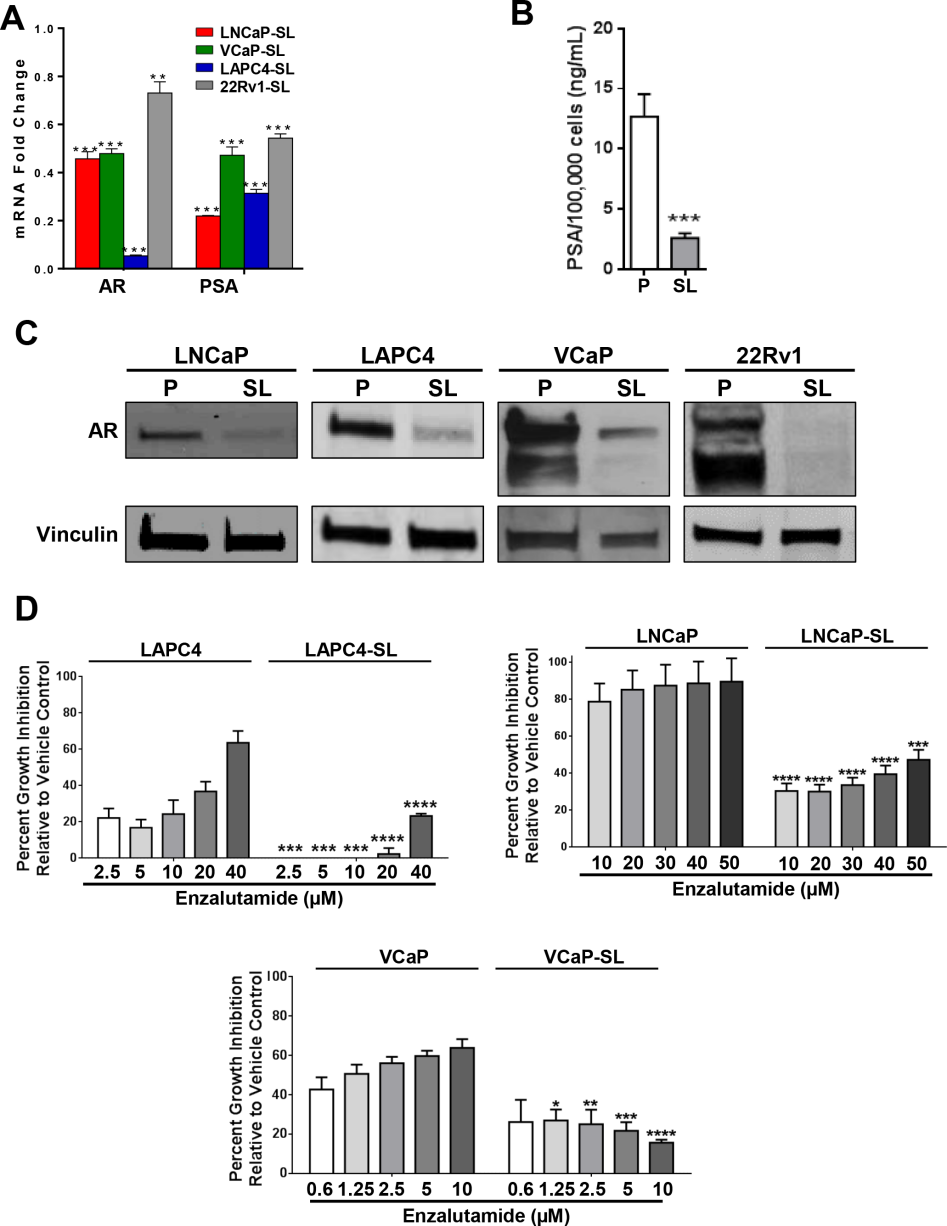
Androgen Receptor axis down-regulation in developmentally reprogrammed PCa cells (**A**) *AR* and *PSA* mRNA expression, measured by RT-qPCR, is down-regulated in reprogrammed (SL) PCa cells compared to parentals. Bars represent relative expressions compared to parental cell lines. Mean ± standard error; **P < 0.01; ***P < 0.001. (**B**) Secretion of PSA into cell medium was significantly downregulated in LNCaP-SL cells over 48 hours compared to parental cells. Mean ± standard deviation; ***P < 0.001. (**C**) Western Blot analyses of parental and reprogrammed PCa cells showed down-regulation of AR and AR-variant protein. (**D**) Relative growth inhibition (7-days) of LAPC4, LNCaP and VCaP parental and stem-like cells with increasing dosages of enzalutamide. Mean ± standard deviation; *P < 0.05; **P < 0.01; ***P < 0.001; ****P < 0.0001.

**Figure 3:**
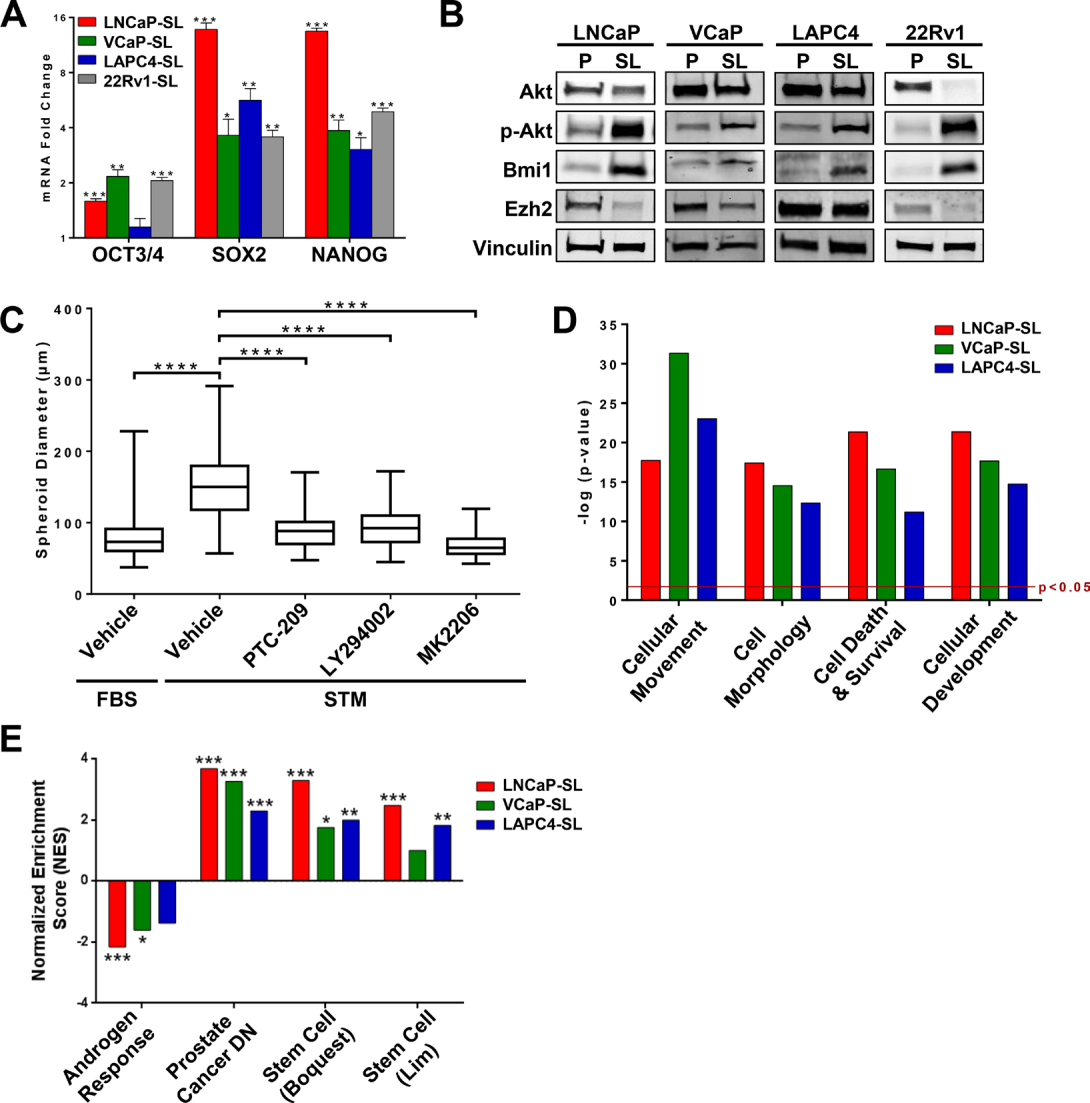
Stem cell-like features of developmentally reprogrammed PCa cells (**A**) Expression of mRNAs for *OCT3/4*, *SOX2* and *NANOG* is increased in reprogrammed PCa cells. Mean ± standard error; *P < 0.05; **P < 0.01; ***P < 0.001. (**B**) Western Blot analyses showed overexpression of BMI1 and phosphorylated-AKT protein in reprogrammed PCa cells. (**C**) Sphere-forming assays indicate significantly smaller spheroid formation of LNCaP cells during developmental reprogramming when AKT and BMI1 pathways are targeted with inhibitors LY294002, MK2206, or PTC209. Mean ± standard deviation; ****P < 0.0001. (**D**) Ingenuity analyses of genes differentially expressed (*p* < 0.05, fold-change > 2.0) in reprogrammed LNCaP, VCaP, and LAPC4 cells indicate that STM-reprogramming induces a significant enrichment of cellular functions related to cancer stem cell phenotypes. (**E**) Gene Set Enrichment Analyses (GSEA) of genes differentially expressed (*p* < 0.05, fold-change > 1.5) in reprogrammed LNCaP, VCaP, and LAPC4 indicate reprogramming is inversely correlated with *Androgen Response* Genes and significantly enriched in *Prostate Cancer-Down Genes* (Liu dataset [56]), and *Stem Cell Genes* (Boquest and Lim datasets [25, 26]).

The changes in morphology and growth patterns coincided with a notable change in gene expression. AR and PSA mRNA and protein expressions were suppressed, including truncated AR-V7 in 22Rv1 cells [3] (Figure 2A–2C). For LAPC4, LNCaP and VCaP cells, this was consistent with a significant loss of sensitivity to growth inhibition by enzalutamide (Figure 2D). Further, evaluation of a small panel of stem cell-associated genes showed that key Yamanaka Factors were commonly overexpressed in all treated PCa cells (Figure 3A). All four reprogrammed cell lines also shared a striking increase in expression of BMI1 and phosphorlyated-AKT protein whereas EZH2 protein expression was reduced or relatively unchanged (Figure 3B). For LNCaP cells, spheroid formation in STM under non-adherent conditions was strongly inhibited by the presence of a BMI1 inhibitor (PTC-209), a PI3-kinase inhibitor (LY294002) or an AKT inhibitor (MK2206) (Figure 3C).

Heat maps of differentially expressed genes (compared to their respective parentals) showed that reprogrammed LNCaP, VCaP and LAPC4 cells had distinct clusters of over- or under-expressed genes that may reflect differing genomic alterations within each cell line (Supplementary Figure 1). The common differentially expressed gene sets, however, were significantly enriched for genes functionally characterized by involvement in *cell development*, *morphology*, *movement* and *cell death and survival* (Figure 3D). On an individual basis, gene set enrichment analysis (GSEA) Hallmarks for *Androgen Response Genes* were significantly reduced while *Prostate Cancer-Down Genes* were significantly enriched in reprogrammed cells (Figure 3E). There was also significant enrichment for *Stem Cell Genes* in the Boquest [25] and Lim [26] stem cell datasets (Figure 3E).

**Figure 4:**
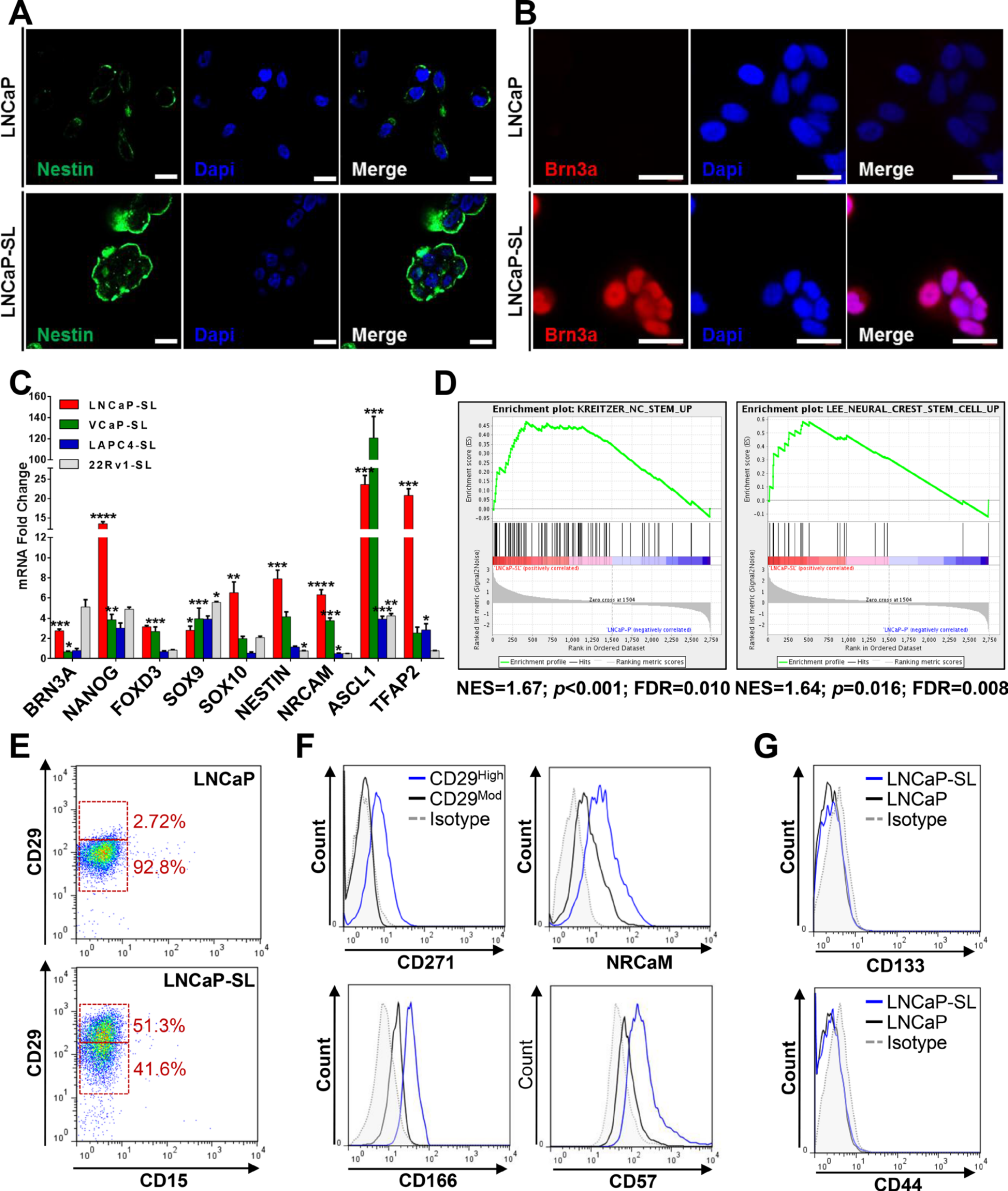
Developmentally reprogrammed PCa cells have characteristics of NC stem cells (**A**) Immunofluorescence staining for NESTIN, a neuroectodermal stem cell marker, in parental and developmentally reprogrammed LNCaP cells. (**B**) Immunofluorescence staining of BRN3A, a neuronal transcription factor, in parental and developmentally reprogrammed LNCaP cells. Scale bars represent 20 μm. (**C**) qPCR analyses indicate that reprogrammed stem-like PCa cells overexpress genes associated with NC stem cells compared to parental cells. Mean ± standard error; *P < 0.05; **P < 0.01; ***P < 0.001; ****P < 0.0001. (**D**) GSEA shows significant enrichment of NC stem cell genes in the Kreitzer and Lee neural crest stem cell datasets [27, 28] in reprogrammed LNCaP cells. (**E**) FACS analyses indicate that reprogramming increases the population of CD29^High^/CD15^−^ cells. (**F**) FACS profiling of CD29^Moderate^ and CD29^High^ LNCaP-SL cells showed that reprogrammed CD29^High^ cells overexpress CD271, NRCaM, CD166 and CD57, surface markers of neural crest stem cells. (**G**) FACS analyses demonstrate that expression of CD133 and CD44 remain unchanged after developmental reprogramming in LNCaP cells.

**Figure 5:**
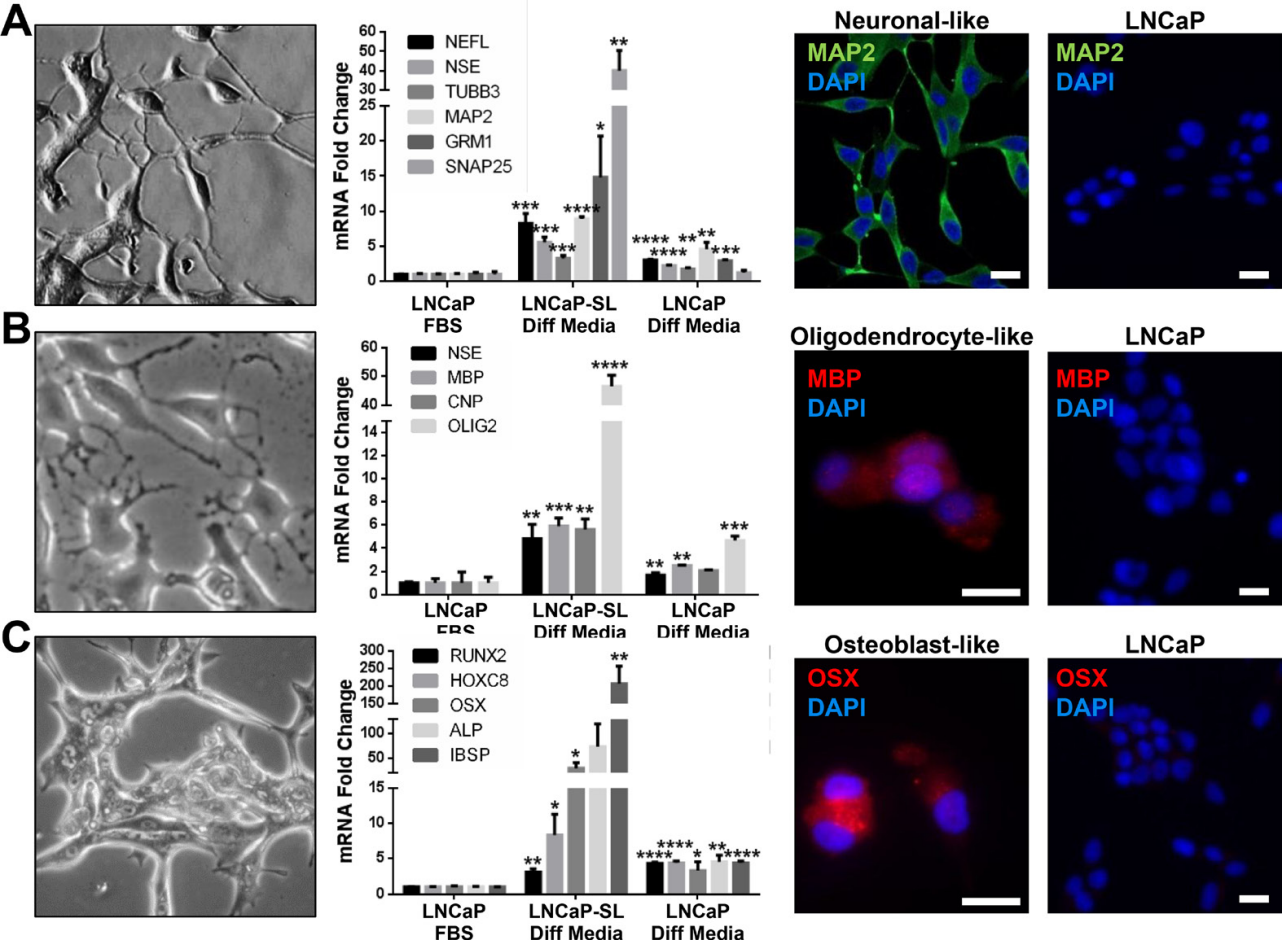
Reprogrammed PCa cells can differentiate to N/NC-derived cell lineages (**A**–**C**) Morphological features and expression of cell lineage biomarkers in LNCaP-SL cells cultured in neuronal (**A**), oligodendrocyte (**B**) or osteoblast (**C**) differentiation mediums (Diff Media) for 14 days, indicating the acquisition of relevant morphology (Left), mRNA expressions (Middle) or protein expressions by IF (Right) indicative of neurons, oligodendrocytes or osteoblasts by LNCaP-SL cells. Mean ± standard error; *P < 0.05; **P < 0.01; ***P < 0.001.

Unique to this model, reprogrammed cells overexpressed genes and proteins associated with N/NC stem cells (Figure 4A–4C). Upregulated genes in reprogrammed LNCaPs were significantly enriched in both the Kreitzer [27] and Lee [28] NC stem cell gene expression databases (Figure 4D). Accordingly, reprogrammed LNCaP cells displayed an altered cell surface marker profile that was congruent with a NC-specific stem cell identity [29] including CD29^High^/CD15^Low^, with increased NRCAM, CD271(NGFR) and CD57(HNK1) expression (Figure 4E, 4F) whereas they lacked expression of surface markers CD133 and CD44 (Figure 4G) that are inconsistent markers of prostate CSCs [30–32]. Similarly, reprogrammed VCaP cells also overexpress N/NC markers CD271/NGFR, NRCaM and CD57/HNK1, as well as CD44 (Supplementary Figure 2). STM-reprogrammed LNCaPs also readily differentiated to NC-derived cell lineages such as neuron-, oligodendrocyte-, osteoblast- or glia-like cells in androgen-free lineage-specific differentiation media, as shown by their representative morphologies as well as by the expression of lineage-specific mRNAs and proteins (Figure 5A–5C, Supplementary Figure 3). Collectively, these characteristics support the idea that STM-driven reprogramming enabled LNCaP conversion to a multipotent N/NC stem-like state.

**Figure 6:**
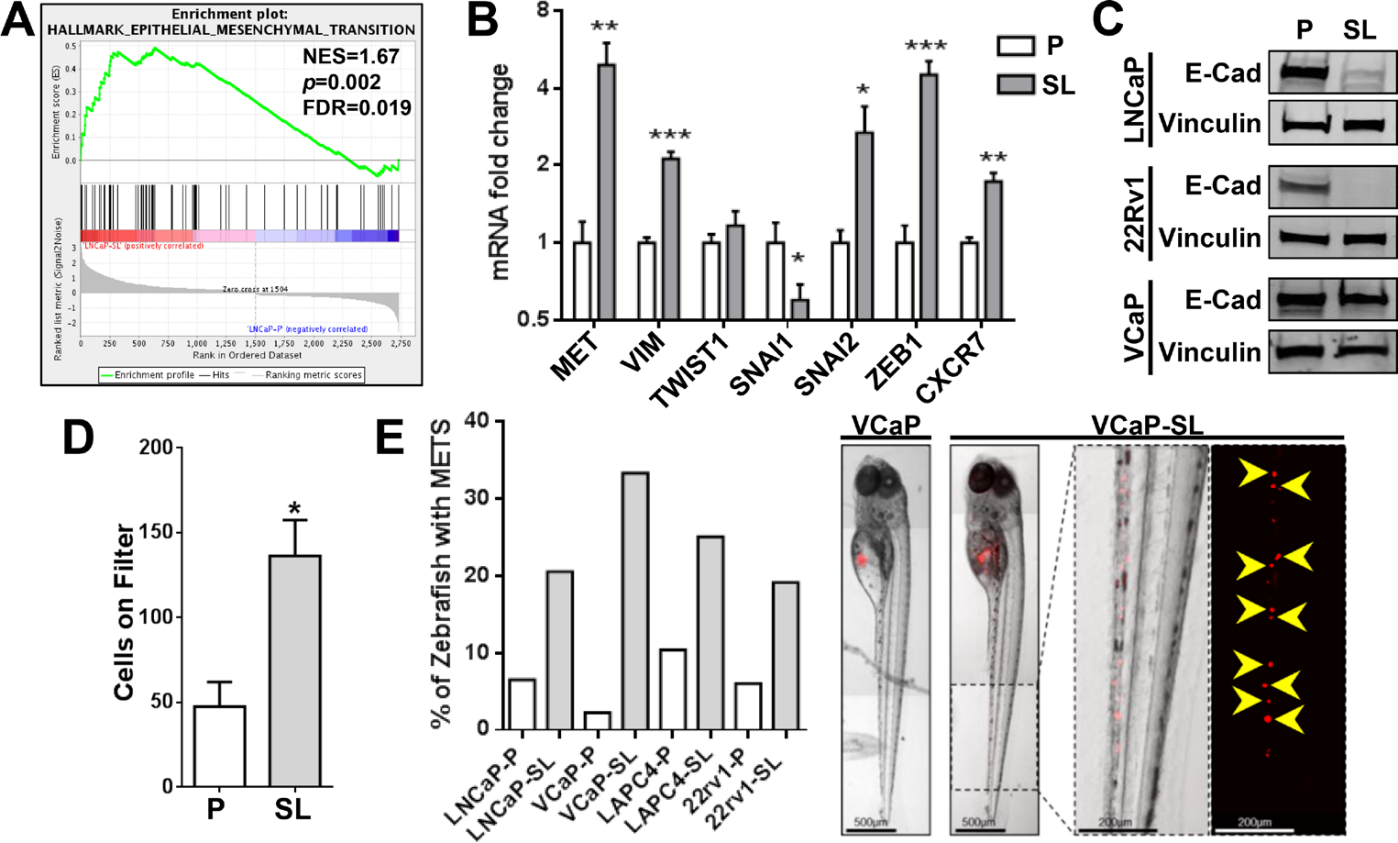
Reprogrammed PCa cells possess increased invasive/metastatic abilities (**A**) GSEA indicates a significant enrichment of EMT genes in LNCaP-SL cells. (**B**) qPCR analyses show that reprogrammed LNCaP-SL cells overexpress mRNA of key EMT markers. Mean ± standard error; *P < 0.05; **P < 0.01; ***P < 0.001. (**C**) Western Blot demonstrates loss of E-Cadherin in reprogrammed PCa cells indicating increased EMT phenotype. (**D**) Reprogrammed LNCaP-SL cells demonstrate increased invasion through a Matrigel-coated membrane in *in vitro* invasion assays compared to parental LNCaP cells. Mean ± standard error; *P < 0.05. (**E**) Reprogrammed stem-like PCa cells are more invasive/metastatic in zebrafish assays (Left). Representative photomicrographs of VCaP and VCaP-SL xenografted fish illustrates dispersal of fluorescently-labelled VCaP-SL cells within the caudal region (Right).

Reprogrammed LNCaPs were significantly more capable of initiating tumors from small numbers of cells xenografted into intact or pre-castrated male mice (Table 1). Tumors formed from reprogrammed (AR^Low^) cells were composed of AR^+^ cells, even in castrates (Supplementary Figure 4). NC stem cells are recognized for their predisposition to EMT and invasion [24, 33]. Thus the overexpression of EMT genes (Figure 6A, 6B) and loss of E-cadherin expression (Figure 6C) in reprogrammed PCa cell lines remains consistent with an invasive NC stem-like phenotype. Accordingly, we observed significantly increased *in vitro* invasion through a Matrigel-coated membrane for developmentally reprogrammed LNCaP cells (Figure 6D). Furthermore, all reprogrammed cell lines displayed increased invasive behaviors *in vivo* in a zebrafish xenograft model compared to parental cells (Figure 6E).

**Table 1:** Subcutaneous tumor xenografts formed from parental or STM-reprogrammed LNCaP cells in intact and castrated nude mice

	Number of Cells Xenografted	Tumors/Xenograft	
		Parental LNCaP	Reprogrammed LNCaP
Intact Nude Mice	2 × 10^6^	4/8	N/A
	1 × 10^6^	5/8	N/A
	5 × 10^5^	0/8	N/A
	1 × 10^5^	0/8	N/A
	1.25 × 10^4^	N/A	4/8
	6.25 × 10^3^	N/A	3/8
	1.25 × 10^3^	N/A	2/8
	1.252 × 10^2^	N/A	1/8
Castrated Nude Mice	1 × 10^6^	3/8	N/A
	1 × 10^5^	0/8	N/A
	5 × 10^4^	0/8	2/8
	1 × 10^4^	0/8	6/8
	1 × 10^3^	N/A	1/8

**Figure 7:**
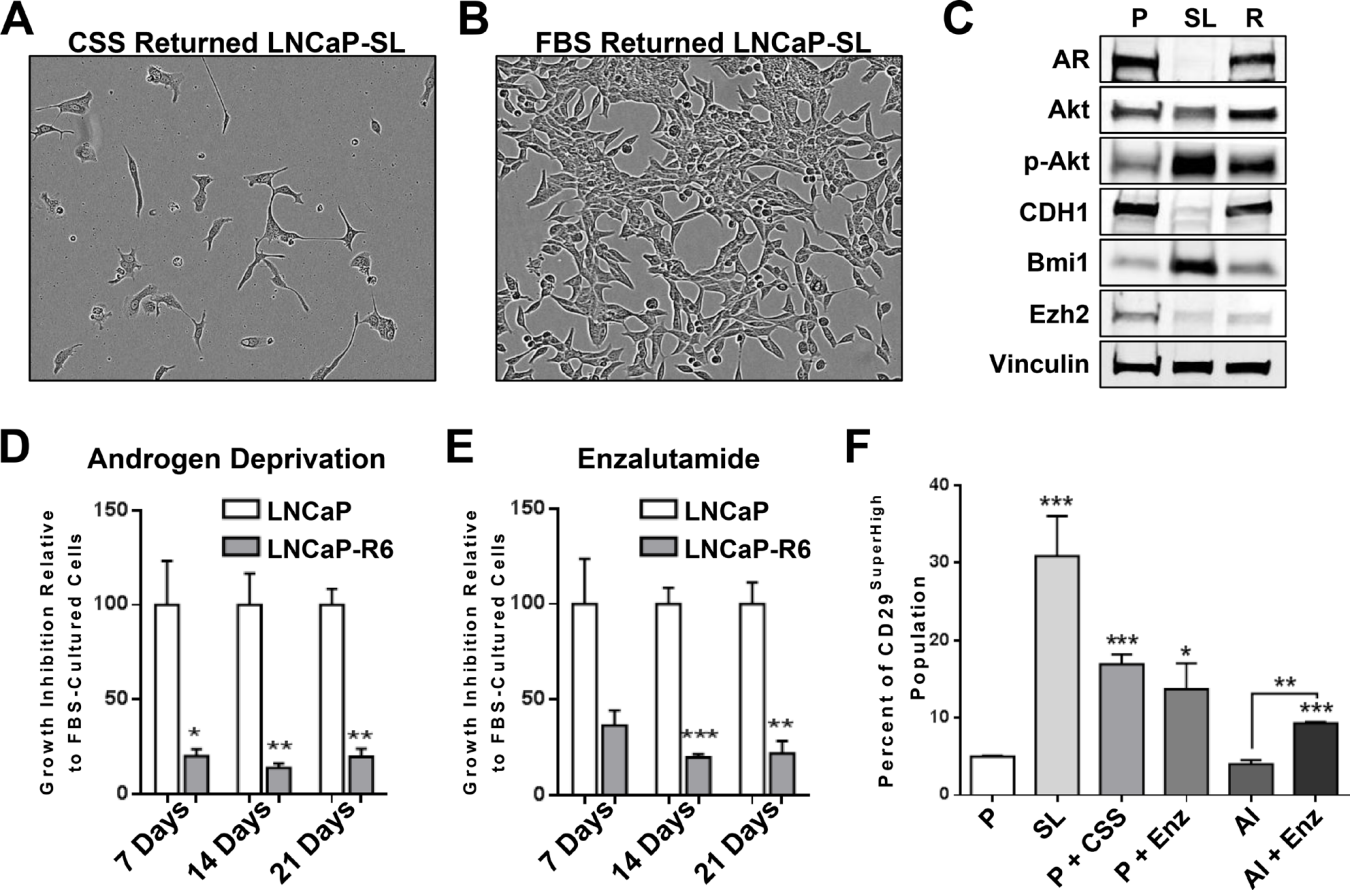
Reprogramming *in vitro* by androgen-deprivation and acquired therapy resistance (**A**) LNCaP-SL cells re-differentiate to neuroendocrine-like cells within 5-days in RPMI with CSS. (**B**) LNCaP-SL cells regain the typical morphology of LNCaP cells within 5-days in RPMI with FBS. Scale bars represents 100 μm. (**C**) Western Blot shows that re-differentiated LNCaP cells regain AR and E-Cadherin expression while retaining higher phosphorylated-AKT and BMI1 protein expression. (**D**) Loss of growth inhibition when LNCaP-R6 cells were grown in RPMI with CSS medium compared to parental LNCaP demonstrates acquired resistance to androgen-deprivation. (**E**) Loss of growth inhibition of LNCaP-R6 cells grown in RPMI with FBS supplemented with 1 μM enzalutamide compared to parental cells demonstrates acquired resistance to enzalutamide. Mean ± standard error; *P < 0.05; **P < 0.01; ***P < 0.001. (**F**) FACS analyses shows the increase of CD29^SuperHigh^ populations in reprogrammed LNCaP cells and in parental LNCaP cultured in RPMI with CSS (P+CSS) or RPMI with FBS and 10 μM enzalutamide (P+Enz) for 3 days, LNCaP androgen-independent cells (AI) cultured in CSS (AI) or in the presence of 10 μM enzalutamide (AI+Enz). Mean ± standard error; *P < 0.05; **P < 0.01; ***P < 0.001.

The metastable nature of STM-reprogrammed cells also allowed re-differentiation to NE-like cells when switched to androgen-free medium (Figure 7A) or return to prostate-like cells with re-expression of AR in medium containing androgen (Figure 7B, 7C, Supplementary Figure 5). We sequentially reprogrammed then re-differentiated LNCaP cells back to prostate-like cells at least 6 times, showing the remarkable plasticity of the phenotypes. By 6 sequential re-cyclings though, AR^+^ “returned” R6 cells showed significant androgen growth-independence, being resistant to both androgen deprivation and enzalutamide (Figure 7D, 7E), despite overexpressing full-length AR protein (data not shown). Finally, acute exposure of parental LNCaP cells to androgen-free medium or enzalutamide increased the population of CD29^High^ cells, (Figure 7F, Supplementary Figure 6A). This population was transient and was lost upon chronic selection under AD to generate androgen growth-insensitive LNCaP-AI cells. LNCaP-AI cells, however, remain sensitive to enzalutamide and acute treatment with enzalutamide also increased the CD29^Hi^/CD133^Lo^/CD44^Lo^ population (Figure 7F, Supplementary Figure 6B, 6C).

### Gene expression changes induced by STM-reprogramming are biologically relevant

**Figure 8:**
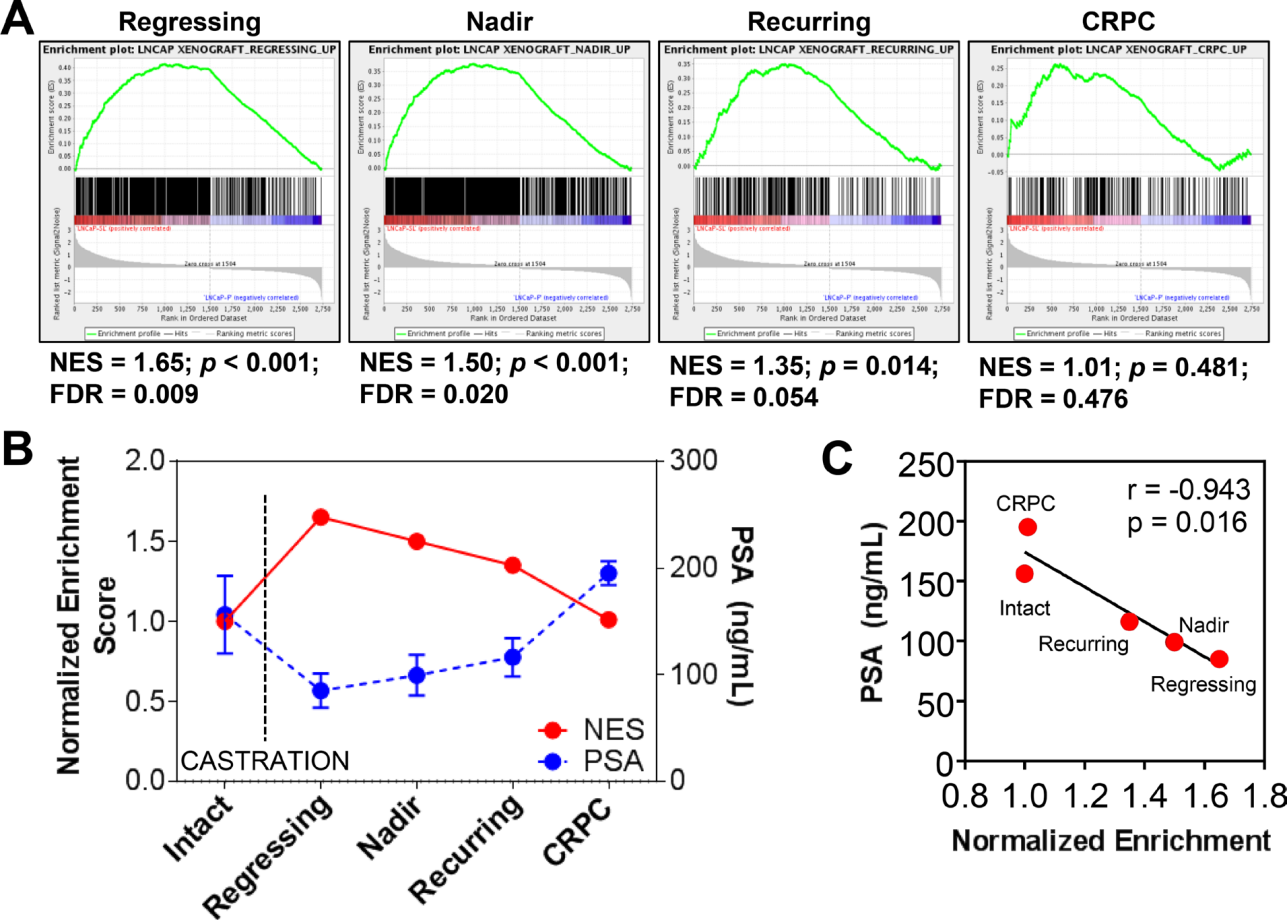
Reprogramming *in vivo* by androgen-deprivation and acquired therapy resistance (**A**–**B**) Genes overexpressed in reprogrammed LNCaP cells are positively enriched in the transcriptomes of LNCaP xenografts after castration of hosts. GSEA-determined normalized enrichment scores shows significant enrichment of stem-like overexpressed genes in RNAs from regressing LNCaP xenografts after castration of the host (*n* = 6) and at PSA nadir (*n* = 9) whereas these genes were not enriched in xenografts prior to castration (*n* = 11) or in recurrent castrate-resistant tumors (*n* = 12). Mean ± standard error. (**C**) The Normalized Enrichment Score (NES) of LNCaP-SL cells compared to LNCaP xenografts before and after castration is inversely and significantly correlated with PSA levels in the blood of murine xenograft hosts.

Gene Set Enrichment Analysis (GSEA) showed that genes overexpressed in reprogrammed LNCaPs (*p* < 0.05, fc > 1.5) were positively enriched in the transcriptomes of LNCaP xenografts following castration of the host mouse (Figure 8A, 8B). Normalized enrichment scores (NES) reach highest significance in regressing and PSA-nadir tumors. Afterwards, NES diminished again to non-significance as tumors relapsed to castration-resistance. Moreover, we observed a reverse correlation of NES with serum PSAs in the xenografts (Figure 8C), suggesting that residual tumor cells at PSA nadir that will repopulate the recurrent tumor are enriched for an mRNA expression profile associated with the *in vitro* reprogramming of tumor cells.

**Figure 9:**
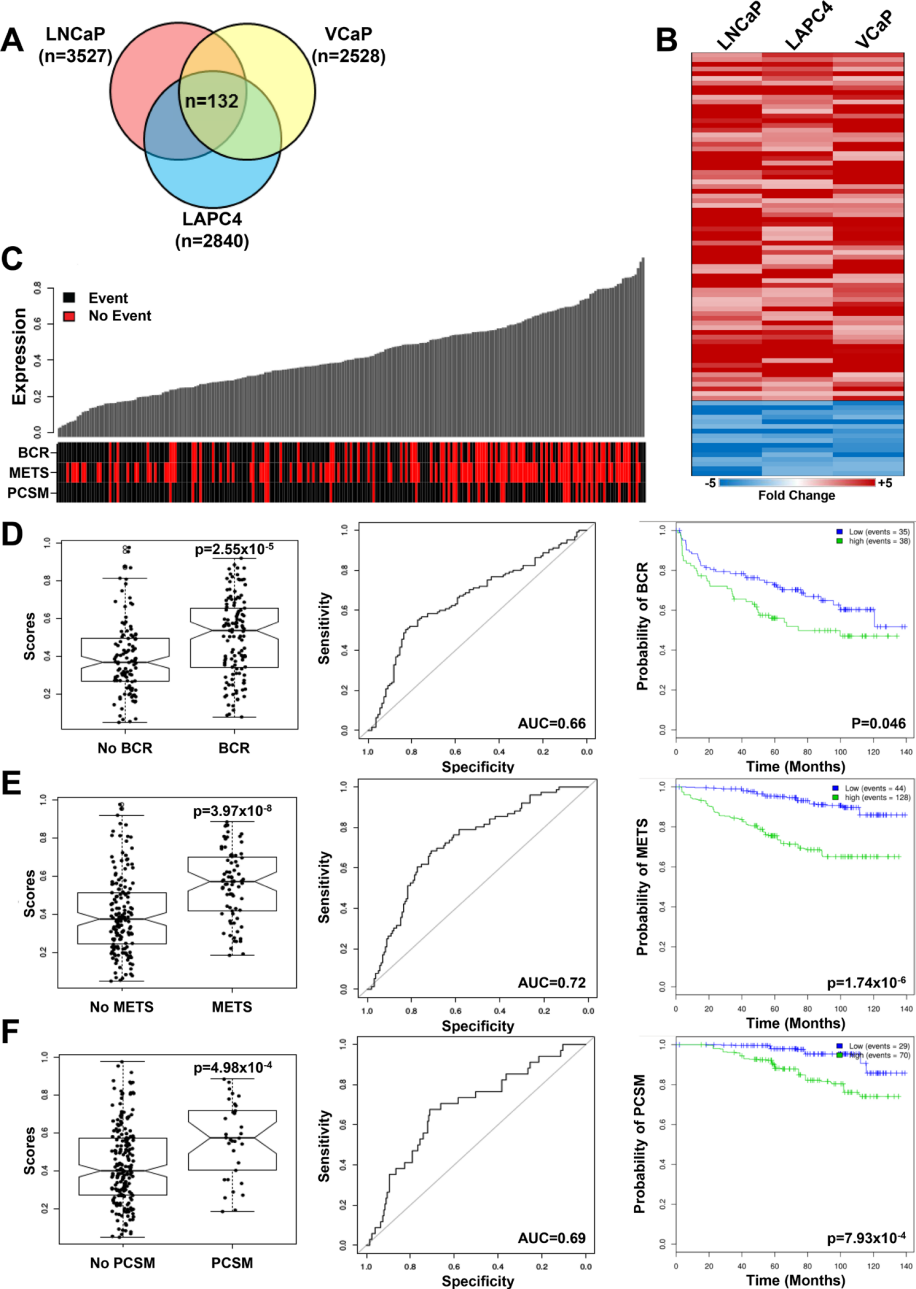
Reprogrammed PCa cells share a gene signature that correlates with adverse outcomes in patients (**A**) Venn Diagram of genes differentially expressed (*p* < 0.05, fold-change > 2) in three reprogrammed PCa cell lines share a common 132 gene signature. (**B**) Heatmap of the top 90 differentially expressed genes (*p* < 0.05, fold-change > 2) in reprogrammed LNCaP, VCaP and LAPC4 PCa cell lines. The complete gene list is found in Supplementary Table 2. (**C**–**F**) Enrichment of the 132 gene reprogrammed PCa cell signature was assessed in the MCII dataset (see supplementary data for results in other patient cohorts) and correlated with PCa patient clinical outcomes, including biochemical recurrence (BCR), development of metastasis (METS) and Prostate Cancer Specific Mortality (PCSM). (**C**) Patient annotation matrix and waterfall plots showing the 132 gene signature score in waterfall plots above a patient annotation matrix. Each column of the waterfall plot relates to a column in the annotation matrix and represents one patient. The rows of the annotation matrix indicate the events which the patients experienced. (**D**) The 132 gene signature score is significantly higher (*p* = 2.55 × 10^−5^, AUC = 0.66) in patients with BCR and a high gene signature score (green line) in primary tumors predicts BCR (*p* = 0.046). (E) The 132 gene signature score is significantly higher (*p* = 3.97 × 10^−8^, AUC=0.72) in patients with METS and a high gene signature score (green line) in primary tumors predicts the development of metastases (*p* = 1.74 × 10^−6^). (**F**) The 132 gene signature score is significantly correlated (*p* = 4.98 × 10^−4^, AUC = 0.69) to prostate cancer specific mortality and a high gene signature score (green line) in primary tumors is significantly correlated with poor prognosis (*p* = 7.93 × 10^−4^).

Finally, global gene expression profiling of three reprogrammed cell lines (LNCaP, VCaP and LAPC4) showed an overlapping set of 132 genes commonly altered by reprogramming, and contained several N/NC stem cell genes including ALCAM(CD166) and SOX9 (Figure 9A, 9B, Supplementary Table 2). Use of this set as a gene signature to analyze clinically-annotated human prostate tumor gene expression datasets showed significant enrichment in patients with biochemical recurrence (BCR) or the presence of overt metastasis (METS) in the Mayo Clinic (MC) I and II, Cleveland Clinic Foundation (CCF) and Memorial Sloan Kettering Cancer Center (MSKCC) datasets (Figure 9C–9F, Supplementary Tables 3, 4). The gene signature was also significantly enriched in tumors from patients with PCa-specific mortality (PCSM) in MCI/II datasets whereas the CCF and MSKCC datasets were not powered for this determination. Relevant Kaplan-Mayer survival curves in the MCII dataset showed that the presence of the gene signature in primary tumors was a significant prognostic marker for BCR, METS and PCSM. This prognostic value was an independent predictor of METS and PCSM in MCII as was shown by its significance in univariant as well as multivariant analyses (in Supplementary Table 4).

## DISCUSSION

Based on re-evaluation of *in vitro* NEtD in LNCaP cells, we proposed the existence of a transient N/NC stem-like state, induced as a consequence of therapy, which mediates NEtD-driven resistance of PCa. We sought to capture and characterize PCa cells in this state with the use of an androgen-free N/NC stem cell medium. Four different AR^+^/PSA^+^ PCa cell lines cultured in STM underwent a similar morphological transition and readily formed spheroids similar to the growth patterns of rare prostate CSCs. These features are strikingly different from *in vitro* NE-transdifferentiated PCa cells in androgen-depleted medium. Additionally, cells continued to grow in STM whereas NEtD *in vitro* results in a growth-quiescence. The use of N/NC stem cell medium was crucial for PCa cell reprogramming; it did not occur when cells were grown in previously reported ESC mediums or with medium supplemented with EGF and bFGF alone, indicating that it is distinct from previously described CSC expansion paradigms where these two supplements often suffice [34]. STM differs from standard mediums used to culture AR^+^ PCa cells in that it lacks serum and androgen and is supplemented with recombinant EGF, bFGF and a commercial neural supplement used in the culture of neural/neural crest stem cells. Due to the proprietary nature of this neural stem cell supplement, we are not privilege to its contents. However we have detected the presence of a neural-derived regulatory protein, Sema3C (data not shown), that may indicate the inclusion of a neural tissue extract. Given reports that PCa cells express a Semaphorin receptor (Plexin B1) and are influenced by Semaphorins [35, 36], perhaps Semaphorin signaling plays some role in the model we described.

Microscopically, we observed virtually the entire population of plated cells undergoing this morphological transformation and clustering. We quantitatively determined reprogramming efficiency for LNCaP cells at approximately 60–80% of the STM-treated parental population, depending on the assay employed. This may be an underestimate since poorly-adherent STM clones were likely lost during medium changes in the assays. Observations on the three other AR^+^ PCa cells switched to STM showed a similar high rate of morphological conversion.

Like prostate CSCs enriched by surface antigen expression or other methods, STM-reprogrammed cells showed markedly reduced expression of AR and PSA. The loss of AR was commensurate with development of resistance to the growth-inhibitory effects of the AR antagonist, enzalutamide. For 22Rv1 and VCaP cells, which overexpress AR-V7, a truncated AR variant, STM-reprogramming suppressed expression of both full-length and AR-V7. Thus, despite the belief that truncated ARs constitutively drive AR signaling, the loss of AR-V7 expression in reprogrammed cells suggest that it is not replacing differentiation functions activated by full-length AR and this is supported by studies contrasting the transcriptome of AR-V7 and full-length AR [37]. STM induced marked upregulation of Yamanaka factors, phosphorylated-AKT and BMI1, thought to represent genes/proteins active in CSCs [38, 39]. BMI1 overexpression is already associated with progression of PCa and acquired resistance to therapeutics [39, 40]. It is part of the polycomb reader complex that plays a role in epigenetic gene silencing and is required for self-renewal of neural and other stem cell types [41]. In contrast to BMI1, EZH2 expression was significantly reduced in most cells by STM-reprogramming. We are currently exploring whether the reduction in EZH2 expression may be a factor in the metastable nature of STM-reprogrammed PCa cells.

**Figure 10:**
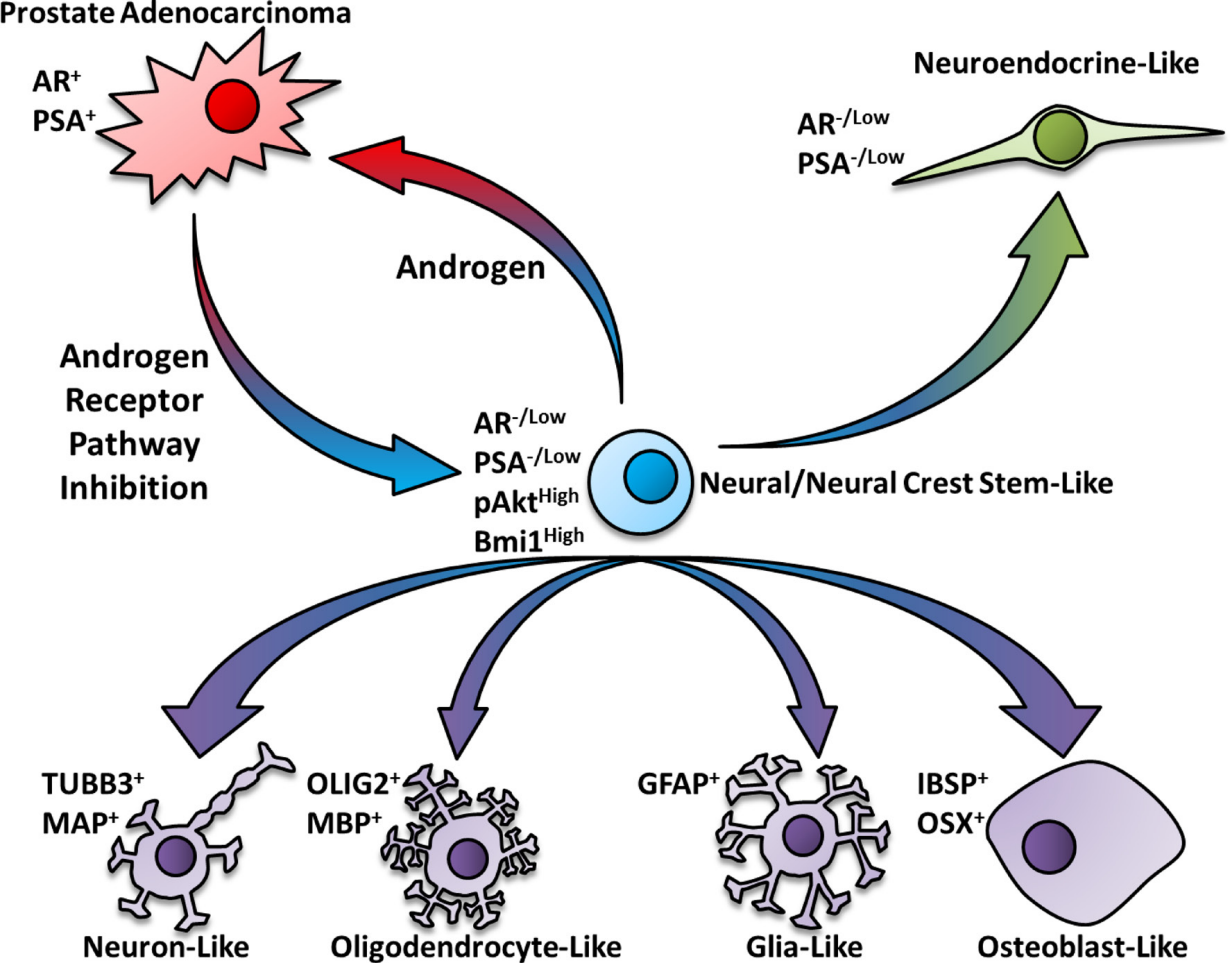
Schematic pathway for the neural/neural crest stem cell-mediated transdifferentiation of prostate cancer cells Based on the outcomes of this study, we propose a model whereby androgen receptor pathway inhibition triggers a developmental reprogramming process that results in a metastable cancer stem-like state that resembles neural/neural crest stem cells. These N/NC stem-like cells may acquire features of differentiated N/NC derived cell lineages when placed into cell-specific differentiation mediums. Depending on the cellular microenvironment, these developmentally reprogrammed cells may transdifferentiate to neuroendocrine-like or other therapy resistant PCa cell types.

Collectively, these changes support the idea that STM triggered a developmental reprogramming process that returned PCa cells to a more stem like state, graphically described in Figure 10. Increased expression of N/NC-specific stem markers (NESTIN, BRN3A, *SOX9*, *SOX10*, *TFAP2A* and *ASCL1*) and cell surface proteins (NRCAM, NGFR, ALCAM, HNK-1 and Integrin-β_1_) on reprogrammed cells addresses the N-/NC-specific stem-like identity of the reprogrammed state as was proposed. Our ability to subsequently direct re-differentiation of reprogrammed LNCaP cells to N/NC-derived cell lineages (neuron, oligodendrocyte, osteoblast and glia) using lineage-specific differentiation mediums further demonstrates their multipotent N/NC stem-like state. Despite the many stem-like characteristics, reprogrammed PCa cells differ from more commonly described CSCs in that they all lacked surface expression of CD133. Whereas reprogrammed LNCaP and LAPC4 cells did not overexpress surface CD44, reprogrammed VCaP and 22Rv1 cells did overexpress it. While the current focus is on CSCs that are marked by CD133 and CD44, our results reinforce incongruous reports of stem cell-like characteristics in cancer cells that lack expression of these antigens [30–32].

Reprogramming also generated a more aggressive PCa cell. Reprogrammed LNCaPs showed high tumor-initiating capacity in both intact and castrated male mice that was in striking contrast to parentals that required at least one million cells to form tumors. Likewise, NC stem cells are known for their invasive abilities during embryonic development and features of EMT [33]. In reprogrammed cells, these EMT features were correlated with an overall enrichment for expression of EMT-related genes, increased expression of *SNAI2*, *ZEB1* and *VIM*, reduced expression of E-cadherin and increased invasiveness *in vitro* and *in vivo*. Accordingly, all four reprogrammed PCa cell lines were significantly more invasive in zebrafish assays than their parental counterparts. This type of developmental plasticity that increases invasiveness and generates neural- and even bone-like phenotypes might facilitate metastatic dissemination of PCa. Perineural invasion at the primary site is a harbinger of metastatic disease and the neural-type plasticity that we observed could facilitate the interaction of PCa cells with nerves [42, 43]. Furthermore, with evidence that PCa cells use neural networks to metastasize to bone, the neural and bone mimicry generated in our *in vitro* system suggests that this plasticity may foster dissemination through nerves and establishment in bone [44–46]. Indeed, our observations that acute treatment with AR inhibitors enabled a transient increase in the population of high Integrin-β_1_ marked cells might explain findings showing that hormonal agents increased metastatic dissemination of PCa [47].

The reprogrammed phenotype was metastable while cells were passaged and maintained in STM. Re-exposure to standard culture medium restored PCa cells to their adherent epithelial state with re-expression of AR. Likewise, AR^Low^ reprogrammed LNCaP cells xenografted into mice formed tumors composed of cells with extensive nuclear AR staining, even in pre-castrates. Despite our interest in the role of NEtD and progression to castration resistance, the vast majority of castration/enzalutamide resistant PCa cells express AR and show indications of continued dependence on AR activity [2]. The castration resistant nature of tumors formed after xenografting of AR^Low^ reprogrammed LNCaP cells led us to study whether *in vitro* cycles of reprogramming followed by re-differentiation could generate an AR^+^ LNCaP variant able to grow in androgen-depleted medium. Indeed, six cycles of repeated reprogramming-redifferentiation yielded AR^+^ androgen growth-independent cells that were also resistant to enzalutamide. As we found that phosphorylated-AKT levels remained elevated in returned AR^+^ PCa cell lines, this resistance may be driven by an AKT-dependent mechanism as also occurs *in vivo* [48].

*In vivo* relevance of the STM reprogramming paradigm is supported by the positive enrichment of genes overexpressed in reprogrammed LNCaP cells in the transcriptomes of LNCaP xenografts only during the acute period after castration. This transient correlation addresses our hypothesis that the reprogrammed phenotype represents an intermediate state leading to resistance (Figure 10). Clinical relevance of the reprogramming paradigm is demonstrated by the ability of the common gene signature of *in vitro* reprogrammed PCa cells to identify patients with adverse outcomes. There is extensive interest in stratifying clinical outcomes of PCa based upon a transcriptomic “classifier” gene signature applied to primary tumors. To date, these predictive signatures were derived from comparisons of individual tumor transcriptomes collected from large patient groups with differing clinical end-points [49, 50]. Our signature, in contrast, was derived from differential gene expression analyses of novel PCa cell models. This correlation suggests that primary tumors from patients with aggressive disease may harbor a stable subset of CSCs that resemble reprogrammed N/NC stem-like cells. Alternatively, such tumors may have a larger population of cells that are cycling between this transitional CSC-like state and a differentiated state, perhaps due to adverse aspects of the tumor microenvironment (hypoxia, low androgen levels).

With regard to therapeutic considerations, based upon the upregulated expressions of phosphorylated-AKT and BMI1 protein in reprogrammed cells, we found that small molecule inhibitors of these proteins effectively blocked clonogenicity and growth in STM. Both of these proteins are already considered to be targets for better control of metastatic CRPC [38–40]. This raises the possibility that their function in CRPC is linked to developmental reprogramming. Collectively, our results suggest that therapy-induced developmental reprogramming provides another mechanism for generating aggressive, therapy-resistant PCa cells. Our findings also support the idea that there may be more than one class of CSC within a prostate tumor and that each may contribute differently to clinical outcomes in PCa patients.

## MATERIALS AND METHODS

### Cell culture and xenografts

LNCaP, VCaP, 22Rv1 (ATCC, Manassas, VA) and LAPC4 cell line (obtained from Dr. Charles Sawyer), were maintained in phenol red-free ATCC-recommended media. Androgen-independent LNCaP cells (LNCaP-AI) [51], were maintained in phenol red-free RPMI1640 with 10% charcoal-stripped serum (CSS). Cell line identifies were established by short tandem repeat profiling (IDEXX). For reprogramming, cells were seeded on plates in standard parental media and, after overnight incubation, were switched to Stem-Transition Medium (STM). STM is formulated from phenol red-free KnockOut DMEM/F12 (Gibco) supplemented with 2 mM GlutaMAX (Invitrogen), 20 ng/mL rhbFGF (Gibco), 20 ng/mL rhEGF (Gibco) and 2% StemPro Neural Supplement (Invitrogen). Medium was changed every 2–3 days. For re-differentiation paradigms, STM-reprogrammed cells were plated onto PEI-coated plates and allowed to attach overnight. Differentiation mediums were then added consisting of phenol red-free Neurobasal medium (Invitrogen) with B-27 Serum-Free Supplement (Invitrogen), 2% CSS, and 2 mM GlutaMAX (Invitrogen), for neuron re-differentation; the same plus 30 ng/mL triiodothyronine (Sigma), for oligodendrocyte re-differentation; phenol-red free D-MEM with 1% N-2 supplement (Invitrogen), 2% CSS, and GlutaMAX, for glia re-differentiation or; low-glucose phenol-red free DMEM, 10% CSS 10 mM B-glycerophosphate, 50 μg/ml ascorbate phosphate, 10 nM dexamethasone, and 10 nM 25-dihydrovitamin D (Sigma), for osteoblast re-differentiation. Re-differentiation to neuroendocrine-like cells employed phenol red-free RPMI-1640 with 10% CSS, and re-differentiation to prostate-like cells used RPMI-1640 with 10% FBS, each for 14 days. For multiple reprogramming/re-differentiation cycles, prostate re-differentiated cells were grown again in STM for 14 days then re-differentiated in FBS containing medium again for a total of 6 cycles to produce therapy resistant LNCaP-R6 cells.

### Mouse xenografting

LNCaP xenografts were established and analysed as previously described [52]. After animals were euthanized, tumors were removed, fixed in formalin and embedded in paraffin before sectioning and immunostaining. Xenografted mice were considered to be “positive” for tumors if the subcutaneous mass was equal to or greater than 150 mm^3^ within 6 weeks of xenografting and consisted of bulk tumor cells. All animal procedures were performed according to the guidelines of the Canadian Council on Animal Care and with appropriate institutional certification.

### Microarray profiling

Gene expression profiling of cells were performed in biological triplicates with an Affymetrix (for LNCaP androgen deprivation) or Agilent (for STM-reprogrammed cells) human gene array platforms. Control parental LNCaP were grown in media supplemented with 10% charcoal-stripped serum (CSS) and 10 pM R1881, while androgen deprived LNCaP cells were cultured for 15 days in media supplemented with CSS alone before RNA extraction. RNAs were labeled using the WT labeling and control kit (Affymetrix) and were hybridized to GeneChip^®^ Human Gene 1.0 ST Arrays. Arrays were scanned using Affymetrix Microarray Scanner System. To compare gene expression between parental PCa cells and STM-reprogrammed cells (LNCaP, VCaP, 22rv1 and LAPC4), RNAs from biological triplicate samples of parental cells (cultured in phenol red-free media with FBS) and corresponding STM-reprogrammed cells (> 14 days in STM) were extracted. Samples for gene expression analysis were prepared following Agilent's One-Color Microarray-Based Gene Expression Analysis Low Input Quick Amp Labeling v6.0. An input of 100ng of total RNA was used to generate Cyanine-3 labeled cRNA. Samples were hybridized on Agilent SurePrint G3 Human GE 8×60K Microarray (AMDID 028004). Arrays were scanned with the Agilent DNA Microarray Scanner at a 3 μm scan resolution and data was processed with Agilent Feature Extraction 11.0.1.1.

### Database accession numbers

Original gene expression microarray datasets have been uploaded to the GEO as public datasets under GSE66851 and. GSE66850.

### Clone formation assay

Approximately 300 LNCaP cells were plated on triplicate polyethylimine (PEI) coated 10cm plates. After 24 hours cells were switched to STM or FBS. Medium was changed every 2–3 days for 14 days. Cells were fixed with 4% Paraformaldehyde (PFA) and stained with 0.05% Crystal Violet (Sigma-Aldrich) for 30 minutes before washing, and counting of colonies.

### Spheroid assays

A single cell suspension of LNCaP was serially diluted to distribute roughly 5–10 cells per well into Ultra Low Adherence Round-Bottom 96-well plates (Corning) in 10% FBS, 10% CSS, or STM. Treatments were conducted 24 hours after plating, and medium/treatments were topped up once every week. Spheroid formation was assessed by microscope imaging after two weeks, with a diameter threshold set at 100 μm to be considered a spheroid. Biological replicate data were analysed statistically by Two-Way ANOVA.

### PSA quantification

PSA concentrations in 48 hour conditioned mediums were determined using the Cobas E411 (Roche Diagnostics) immunoassay system following manufacturer's instructions. PSA concentrations were normalised to the number of cells on the dish at the time of media harvest. Biological replicate data were analysed statistically by pair-wise Student's *T*-test.

### Western blotting

Cell lysates were prepared in RIPA buffer with protease and phosphatase inhibitors. Approximately 30μg to 50μg of protein were run on pre-cast 4–15% gradient gels (BioRad), and transferred to PVDF membranes. Detection was performed using peroxydase-conjugated and fluorescently-conjugated secondary antibodies. See Supplementary Materials and Methods for details of antibodies employed.

### Quantitative real-time PCR

Total RNA was isolated using the RNeasy RNA Isolation Kit (Qiagen) before reverse transcription with Maxima kit (Thermo Scientific). Subsequent qPCR was performed using an ABI-ViiA7 with SYBR-Green detection (Applied Biosystems). Gene expression was normalized to the housekeeping gene *RPL32*. See Supplementary Table 5 for primer sequences employed for PCR amplification. Biological replicate data were analysed statistically by pair-wise Student's *T*-test.

### Proliferation assays

Cells were plated at 2000 cells/well in quadruplicate on a 96-Well Plate (Corning) on day -1. Experimental and control treatments were conducted and initial readings taken on day 0. CyQuant Direct Cell Proliferation Assay (Invitrogen) was employed for determination of live cell DNA content. Biological replicate data were analysed statistically by Two-Way ANOVA.

### Gene set enrichment analysis (GSEA)

The GenePattern server (http://www.genepattern.org/), and default parameters, were employed to identify significantly-enriched gene signatures in reprogrammed PCa cells (*p* < 0.05, fold change > 1.5). The gene sets used for enrichment were obtained from the “Curated” and “Hallmark” MSigDB collections. Custom gene sets, including the Kreitzer Neural Crest Stem Cell (GSE37859) and LNCaP xenograft progression series (GSE44319) were extracted from GEO and uploaded to GSEA as gene matrix files (.gmx).

### Flow cytometry

Cells were detached by treatment with Accutase (Gibco), washed, incubated for 10 minutes with Human Fc-Blocker (eBioscience), then incubated with target antibodies for 30 minutes at 4°C. Cells were washed again and analysed on a FACSCanto™ II (BD Biosciences). See Supplementary Materials and Methods for details of antibodies employed. Data was analyzed using FlowJo software (TreeStar, USA). Biological replicates were analysed statistically by Two-Way ANOVA.

### Immunofluorescence

For LNCaP and STM-reprogrammed LNCaP, cells were plated at 25,000 cells per well in 4 chamber slides coated with PEI. Cell were incubated for 48 hours before fixing in 1% PFA. Slides were washed once with PBS before permeabilization with 0.5% Triton (30 minutes at room temperature). Cells were washed again before blocking in 3% milk for one hour, and overnight incubation with BRN3A and NESTIN antibodies. Cells were washed briefly before 1 hour incubation with secondary antibodies, and mounted in DAPI mounting media (Sigma). For differentiation to N/NC lineages, 25,000 LNCaP-SL cells were plated in PEI coated 4 chamber slides and allowed to attach for 24 hours before medium was replaced with osteoblast, neuron, glial, or oligodendrocyte re-differentiation medium. Media was refreshed every 3 days, and at day 8 cells were fixed and stained as described above. Immunofluorescence imaging was carried out on an Axio Observer Z1 Microscope (Carl Zeiss). See Supplementary Materials and Methods for details of antibodies employed.

### Immunohistochemical staining

Immunohistochemical staining of tumor xenograft sections and spheroid sections was done on a Ventana autostainer model Discover XT (Ventana Medical System, Tuscan, Arizona) with an enzyme labeled biotin streptavidin system and solvent resistant DAB Map kit using corresponding primary antibody. For each biomarker, representative cores (clearly positive, clearly negative and mixed positive/negative) were manually identified by Dr. Ladan Fazli MD, Pathologist.

### Invasion assay

20,000 LNCaP and LNCaP-SL cells were seeded in the top chamber of a matrigel-coated 24-well plate inserts (Corning) in serum-free medium. 10% FBS was added to the lower chamber as a chemo-attractant. After 20 hours, cells were fixed and stained with DAPI, the filter was fluorescently imaged and cells remaining on the filter counted.

### Zebrafish metastasis assay

A wildtype zebrafish strain was maintained in aquaria according to standard protocols under approval of the Institutional Animal Care Committee. Embryos were dechorionated at 2 days post-fertilization. Parental and reprogrammed PCa cells were fluorescently labelled the day before microinjection with 1.5 μM of CellTracker CM-Dil dye (Life Technologies) per manufacturer's instructions. Following anaesthetisation with Tricaine (Sigma-Aldrich), approximately 50–70 cancer cells were microinjected into the yolk sac of each fish. Embryos were then transferred to 100 mm^2^ plates that contained aquaria water with added phenylthiourea to prevent pigment formation. Approximately 50 fish were injected per cell line, and fluorescent cell dispersal throughout the fish was determined by observation using the Zeiss Axio Observer microscope at 5X objective. Images were then analyzed with Zen 2012 software.

### Correlation with cohorts of PCa patients

The 132-gene signature of reprogrammed PCa cells was correlated with clinically-annotated cohorts of PCa patients in the Mayo Clinic (MCI and MCII) [49, 53], Cleveland Clinic Foundation (CCF) [54], and the Memorial Sloan Kettering (MSKCC) [55] patient cohorts. Statistical analyses were performed in R, version 3.2.2 and all statistical tests were two-sided using a 5% significance level. To test the significance of the association with outcome (biochemical recurrence, metastases, and prostate specific cancer mortality), Wilcoxon rank sum were used. The classifier was constructed using the glmnet package (glmnet 2.0–2). MCII Kaplan-Meier curve *p*-values were generated with a weighted Cox regression model (survival 2.38–3). Detailed methodology for patient correlations are found in the Supplementary Materials and Methods.

## SUPPLEMENTARY MATERIALS FIGURES AND TABLES






