# Supplementary material for: Therapy-induced developmental reprogramming of prostate cancer cells and acquired therapy resistance
Source: Oncotarget. 2017 Jan 27;8(12):18949–67. doi: 10.18632/oncotarget.14850 (PMC5386661; doi:10.18632/oncotarget.14850)
Supplement: Supplementary file 1 [file oncotarget-08-18949-s001.pdf]

Therapy-induced developmental reprogramming of prostate cancer cells and acquired therapy resistance

Supplementary Materials

Antibodies and reagents

The antibodies and concentrations used for immunostaining, and Western blotting were: anti-BRN3A (Millipore, AB5945, 1:500), anti-NESTIN (Millipore, MAB5326, 1:200), anti-MAP2 (Millipore, MAB3418, 1:500), anti-MBP (Abcam, ab40390, 1:500), anti-OSX (Abcam, ab22552, 1:500), anti-GFAP (DAKO, z0334, 1:200), anti-CD133 (Miltenyi Biotec, 130-090-826, 1:10 and 130-090-854, 1:5), anti-CD44 (eBioscience, 17-0441-81, 1:10), anti-CD56 (eBioscience, 17-0567, 1:10), anti-CD29 (eBioscience, 12-0299-42 and 17-0288-41, 1:10), anti-CD15 (eBioscience, 48-0159-41, 1:10), anti-CD271 (BioLegend, 345103, 1:5), anti-NRCAM (Abnova, MAB3561, 1:20), anti-CD166 (eBioscience, 46-1668-41, 1:10), anti-CD57 (BioLegend, 359607, 1:10), anti-AR (N-20) (Santa Cruz, sc-816, 1:1000), anti-AR (441) (Santa Cruz, sc-7305, 1:1000), anti-E-Cadherin (BD Bioscience, 610181, 1:10000), anti-Vinculin (Sigma-Aldrich, V9131, 1:5000), anti-EZH2 (BD Bioscience, 612666, 1:1000), anti-AKT (Cell Signaling, 2920S, 1:1000), anti-phosphorylated-AKT (Cell Signaling, 3787S, 1:1000), and anti-BMI1 (Santa Cruz, sc-10745, 1:200). Enzalutamide was obtained from MedChem Express (NJ, USA). LY294002, PTC209, MK2206 were obtained from SelleckChem (TX, USA).

Gene expression analysis

Processed signal was quantile normalized with Agilent GeneSpring 12.0. Gene clustering and Venn diagram were also generated using Agilent GeneSpring 12.0 to determine the 132 gene signature common to all three cell lines. Only genes with a fold-change > 2 and p-value < 0.05 were considered. This produced a list of 150 commonly altered genes, which contained 132 probe overlap with the Mayo Clinic II (MCII), The Cleveland Clinic Foundation (CCF) Memorial Sloan Kettering (MSKCC) cohort datasets. Cellular and Molecular functions enrichment in STM-reprogrammed PCa cells was assessed using Ingenuity Pathway Analysis (IPA®, QIAGEN) by comparing the imported microarray data generated from our PCa cell lines with the Ingenuity® knowledge base. A list of relevant networks, canonical pathways and algorithmically generated mechanistic networks based on their connectivity was obtained. A score (p-score = −log10[p-value]) according to the fit of the set of supplied genes and a list of biological functions stored in the Ingenuity Knowledge Base are generated. P-scores > 1.3 are significant. Only genes with a fold-change > 2 and p-value < 0.05 were considered.

Association of 132 gene signature with patients' cohort dataset

Patient Cohorts

The Mayo Clinic I (MCI) cohort was designed as a case-cohort study as described previously [1, 2]. In this study men are matched triples of metastatic progression (N = 213), biochemical recurrence after prostatectomy (N = 213) and patients with no evidence of disease (N = 213). The Mayo Clinic II (MCII) cohort is a case-cohort study consisting of a cohort of 1010 men at high-risk for recurrence after receiving radical prostatectomy between 2001–2006 [2–4]. The Cleveland Clinic Foundation (CCF) patient cohort is a case-control study with a 1:3 ratio of metastatic cases (N = 53) versus non-metastatic cases (N = 144) as described previously [2, 5]. These patients were sampled from a total of 2,641 men that were conservatively treated after undergoing radical prostatectomy at Cleveland Clinic between 1987–2008. The Memorial Sloan Kettering (MSKCC) patient cohort has already been described [6].

Specimen selection and processing

After histopathologic review of formalin-fixed paraffin embedded (FFPE) tumor blocks from each case by two expert GU pathologists, the tumor block with the highest Gleason score, regardless of tumor volume was selected for specimen processing. Two x0.6 mm diameter biopsy punch tool cores were used to sample the primary Gleason grade of the index lesion and placed in a microfuge tube for RNA extraction. RNA extraction and microarray hybridization was performed

using clinical-grade techniques in a Clinical Laboratory Improvement Amendments (CLIA)-certified laboratory facility (GenomeDx Biosciences, San Diego, CA, USA). CLIA certification was obtained through the Centers for Medicare and Medicaid Services through standard procedures, and laboratory facilities satisfied all criteria required for certification. Total RNA was extracted and purified as described previously [3]. Following microarray quality control using the Affymetrix Power Tools packages, probeset normalization and summarization was performed using the Single Channel Array Normalization (SCAN) algorithm, which normalizes each sample individually by modeling and removing probe- and array-specific background noise [7]. To calculate gene expression, we used Affymetrix Core level summaries for annotated genes. All RNA and micorarray processing was performed in a CLIA-certified laboratory.

Microarray data are available on the NCBI Gene Expression Omnibus as accession numbers GSE46691 (Mayo Clinic I), GSE62116 (Mayo Clinic II), GSE62667 (Cleveland Clinic) and GSE21032 (MSKCC).

Statistical analysis

Metastatic progression after prostatectomy was defined as a positive CT scan or bone scan. Secondary aims were to assess the prognostic value of the signature for biochemical recurrence and death from prostate cancer. A classifier to distinguish between metastatic vs non-metatstatic cancers was constructed from the 132 gene signature, as a generalized linear model with elastic net regularization. Statistical analyses were performed in R, version 3.2.2 and all statistical tests were two-sided using a 5% significance level. To test the significance of the association with outcome, Wilcoxon rank sum were used. The classifier was constructed using the glmnet package (glmnet 2.0-2). A 10-fold cross-validation was used to tune the lambda value used in glmnet. The final classifier outputs a continuous variable score ranging between 0 and 1, with a higher score indicating a higher probability of metastasis. The classifier model was generated using the Mayo Clinic I cohort as training data. Samples in this cohort were assigned into training (n = 359) and validation (n = 186) as described in Erho 2013. The classifier model was trained on the training portion and the remainder was used for testing. Classifier scores were subsequently generated for every sample in the validation cohorts and performance was assessed separately in each cohort using area under receiver operating characteristic (AUC) curves (pROC_1.8). MCII Kaplan-Meier curve p-values were generated with a weighted Cox regression model (survival 2.38-3).

REFERENCES

1. Nakagawa T, Kollmeyer TM, Morlan BW, Anderson SK, Bergstralh EJ, Davis BJ, Asmann YW, Klee GG, Ballman KV, Jenkins RB. A tissue biomarker panel predicting systemic progression after PSA recurrence post-definitive prostate cancer therapy. PLoS One. 2008; 3:e2318.
2. Prensner JR, Zhao S, Erho N, Schipper M, Iyer MK, Dhanasekaran SM, Magi-Galluzzi C, Mehra R, Sahu A, Siddiqui J, Davicioni E, Den RB, Dicker AP, et al. RNA biomarkers associated with metastatic progression in prostate cancer: a multi-institutional high-throughput analysis of SChLAP1. Lancet Oncol. 2014; 15:1469–1480.
3. Karnes RJ, Bergstralh EJ, Davicioni E, Ghadessi M, Buerki C, Mitra AP, Crisan A, Erho N, Vergara IA, Lam LL, Carlson R, Thompson DJ, Haddad Z, et al. Validation of a genomic classifier that predicts metastasis following radical prostatectomy in an at risk patient population. J Urol. 2013; 190:2047–2053.
4. Prensner JR, Iyer MK, Sahu A, Asangani IA, Cao Q, Patel L, Vergara IA, Davicioni E, Erho N, Ghadessi M, Jenkins RB, Triche TJ, Malik R, et al. The long noncoding RNA SChLAP1 promotes aggressive prostate cancer and antagonizes the SWI/SNF complex. Nat Genet. 2013; 45:1392–1398.
5. Erho N, Crisan A, Vergara IA, Mitra AP, Ghadessi M, Buerki C, Bergstralh EJ, Kollmeyer T, Fink S, Haddad Z, Zimmermann B, Sierocinski T, Ballman KV, et al. Discovery and validation of a prostate cancer genomic classifier that predicts early metastasis following radical prostatectomy. PLoS One. 2013; 8:e66855.
6. Taylor BS, Schultz N, Hieronymus H, Gopalan A, Xiao Y, Carver BS, Arora VK, Kaushik P, Cerami E, Reva B, Antipin Y, Mitsiades N, Landers T, et al. Integrative genomic profiling of human prostate cancer. Cancer Cell. 2010; 18:11–22.
7. Piccolo SR, Sun Y, Campbell JD, Lenburg ME, Bild AH, Johnson WE. A single-sample microarray normalization method to facilitate personalized-medicine workflows. Genomics. 2012; 100:337–344.

Supplementary Table 1: Annotated list of over-expressed Neural/Neural Crest (N/NC) related genes in LNCaP cells cultured 15 days in androgen deprived medium. See Supplementary_Table_1

Supplementary Table 2: List of the developmentally reprogrammed PCa cell line derived 132-gene signature. See Supplementary_Table_2

Supplementary Table 3: Clinicopathological characteristics of all datasets

	Variable	MCI (*n* = 545)	MCII (*n* = 235)	CCF (*n* = 182)	MSKCC (*n* = 131)
**BCR**	no	157	110	130	104
	yes	388	125	52	27
**METS**	no	333	159	133	122
	yes	212	76	49	9
**PCSM**	no	413	201	168	131
	yes	132	34	13	0
	NA	0	0	1	0

BCR (biochemical recurrence); METS (metastases); PCSM (prostate cancer specific mortality); n (number of patients); MC (Mayo Clinic); CCF (Cleveland Cancer Foundation); MSKCC (Memorial Sloan Kettering).

Supplementary Table 4: Summary of the 132 gene signature *p*-values in patient cohorts.

Patient Cohorts	Clinical Outcomes	Wilcoxon Test	Univariable Analysis	Multivariable Analysis	Kaplan Meier
MCI	BCR	1.86 × 10-7	2.27 × 10-6	1.33 × 10-3	NA
	MET	6.78 × 10-24	3.93 × 10-18	9.58 × 10-10	NA
	PCSM	6.98 × 10-22	8.37 × 10-16	1.83 × 10-8	NA
MCII	BCR	2.55 × 10-5	4.77 × 10-2	9.83 × 10-2	4.58 × 10-2
	MET	3.97 × 10-8	1.74 × 10-6	9.04 × 10-4	1.74 × 10-6
	PCSM	4.98 × 10-4	7.93 × 10-4	2.95 × 10-2	7.93 × 10-4
CCF	BCR	7.43 × 10-8	1.20 × 10-5	1.56 × 10-2	NA
	MET	3.50 × 10-7	6.22 × 10-5	7.34 × 10-2	NA
	PCSM	NA	NA	NA	NA
MSKCC	BCR	1.09 × 10-2	1.23 × 10-1	9.65 × 10-1	NA
	MET	1.47 × 10-3	3.99 × 10-2	1.35 × 10-1	NA
	PCSM	NA	NA	NA	NA

MC (Mayo Clinic); CCF (Cleveland Cancer Foundation); MSKCC (Memorial Sloan Kettering Cancer Center); BCR (biochemical recurrence); METS (metastases); PCSM (prostate cancer specific mortality); ns (non significant): NA (not available).

Supplementary Table 5: Primer sequences employed in qPCR experiments in order of appearance in manuscript

Gene	Forward (5′-3′)	Reverse (5′-3′)
RPL32	CCCCTTGTGAAGCCCAAGA	GACTGGTGCCGGATGAACTT
AR	CTGGACACGACAACAACCAG	CAGATCAGGGGCGAAGTAGA
KLK3 (PSA)	AGTGCGAGAAGCATTCCCAAC	CCAGCAAGATCACGCTTTTGTT
OCT3/4	CCCTGGTGCCGTGAAGCTGGAGAAGG	TACTGGTTCGCTTTCTCTTTCGGGCCTG
SOX2	TACAGCATGTCCTACTCGCAG	GAGGAAGAGGTAACCACAGGG
NANOG	CAATGGTGTGACGCAGGTAA	GGTTGCTCCACATTGGAAGG
POU4F1 (BRN3A)	CGGACTTTCGGAGTGTTTGTG	TACTTGTGCTCAGGGAGGGT
FOXD3	CAAGAACAGCCTAGTGAAGC	CCGAAGCTCTGCATCATGAG
NESTIN	CAGGGGCAGACATCATTGGT	CACTCCCCCATTCACATGCT
NRCAM	TTGTGCAAAGAGGGAGCATG	GGGCAGTTCCCTGTTGTCCT
ASCL1	CCCAAGCAAGTCAAGCGACA	CGTTTGCAGCGCATCAGTTC
TFAP2A	ACGCCGATCCATGAAAATGC	CGTTGACGTGGGAGTAAGGA
NEFL	TCCTACTACACCAGCCATGT	TCCCCAGCACCTTCAACTTT
NSE	TCTGCAGTCCCGAGATCCCAGC	CTGATGAGGGCTGGCGCGAT
TUBB3	AGGACGGACAGACCCAGAC	CTGATGACCTCCCAAAACTTG
MAP2	CAGGAATTGACTCCCTCTACAGC	TCTTCACCAGGCTTACTTTGC
GRM1	AGACCAATGAGACGGCCTG	CCTCCTCTACGTTGTAAAGGGT
SNAP25	AGTTGGCTGATGAGTCGCTG	TGAAAAGGCCACAGCATTTC
MBP	TTAGCTGAATTCGCGTGTGG	GAGGAAGTGAATGAGCCGGTTA
CNP	GGCCACGCTGCTAGAGTGCAAGAC	GGTACTGGTACTGGTCGGCCATTT
OLIG2	CGACTCATCTTTCCTTCTCTAA	CGCACTTACCTCATCATTG
RUNX2	CCTCCTACCTGAGCCAGATG	ATGAAATGCTTGGGAACTGC
HOXC8	ATGCCCAGCATACACTCTCTTGT	ATAAATACCAGAGAAGCACCGTGAA
OSX	AGCGACCACTTGAGCAAACAT	GCGGCTGATTGGCTTCTTCT
ALP	TGACCTTCTCTCCTCCATCC	CTTCCTGGGAGTCTCATCCT
IBSP	AAAGTGAGAACGGGGAACCT	GATGCAAAGCCAGAATGGAT
MET	AGGTGTTGGGAAAAAGGTGA	ATTCAGCTGTTGCAGGGAAG
TWIST1	CACTGAAAGGAAAGGCATCA	GGCCAGTTTGATCCCAGTAT
SNAI1	CCTCCCTGTCAGATGAGGAC	CCAGGCTGAGGTATTCCTTG
SNAI2	AGATGCATATTCGGACCCAC	CCTCATGTTTGTGCAGGAGA
CXCR7	GCAGAGCTCACAGTTGTTGC	GCTGATGTCCGAGAAGTTCC
Primers for *ZEB1*, *VIM*, *SOX9* and *SOX10* were purchased directly from Qiagen		

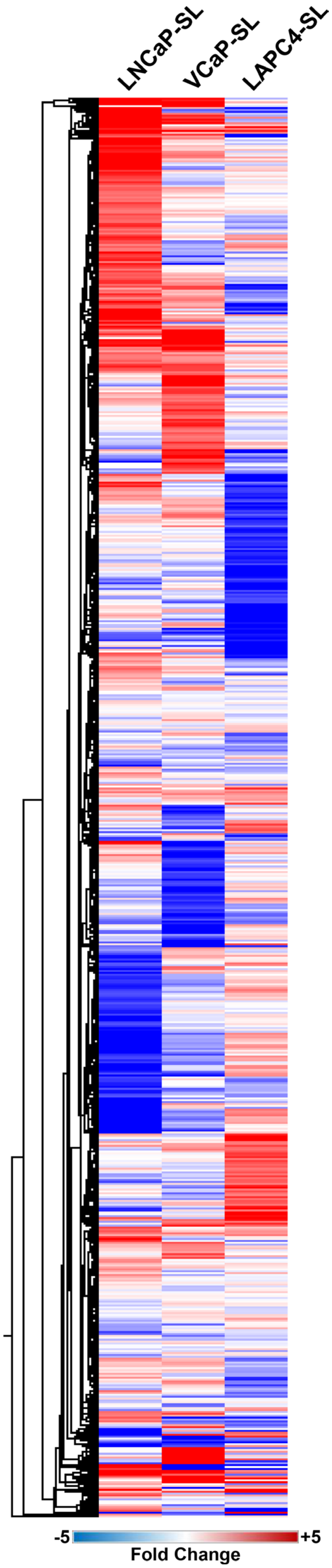

Supplementary Figure 1: Distinct clusters of over-/under-expressed genes in reprogrammed PCa cells. HeatMap of genes up- or down-regulated compared to parentals from 3 different reprogrammed PCa cell lines, as indicated, showing distinct clusters of genes over-/under-expressed in each ($p > 0.05$, fold-change > 2.0).

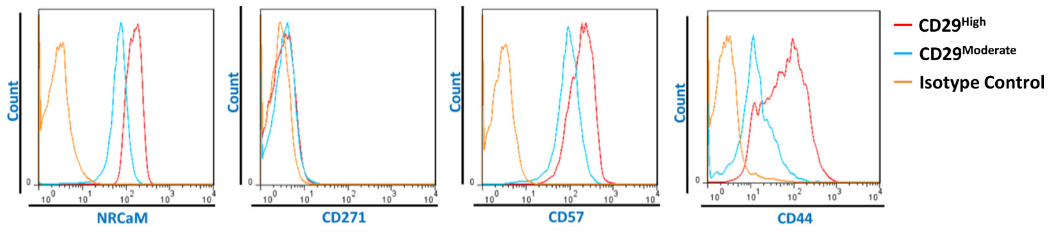

Supplementary Figure 2: Developmentally reprogrammed VCaP cells displayed increased expression of N/NC specific stem cell surface markers. Like LNCaP-SL cells, VCaP-SL cell surface profile is congruent with a CD29^{High}/CD15^{Low} phenotype, and the CD29^{High} population is also strongly correlated with NRCAM, CD271(NGFR) and CD57 (HNK1) expression.

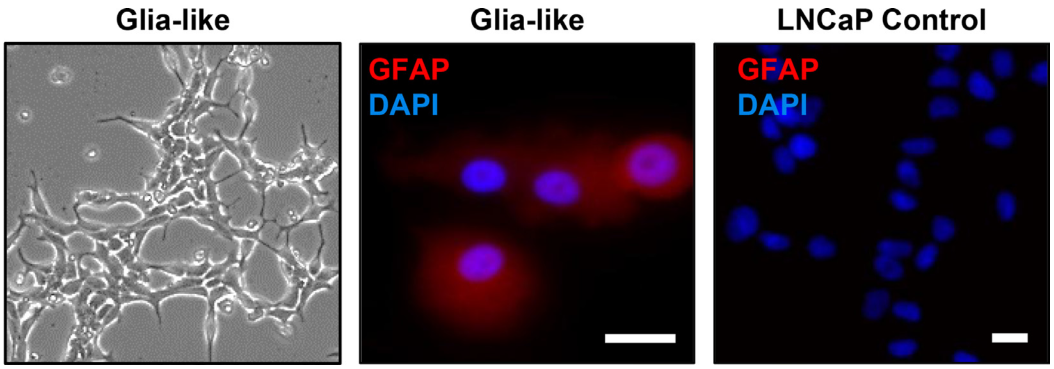

Supplementary Figure 3: Developmentally reprogrammed LNCaP cells can re-differentiate in vitro to Glia-like cells. Morphological features and expression of cell lineage biomarkers by reprogrammed LNCaP cells cultured in glial re-differentiation mediums for 14 days. Phase contrast imaging (Left) or immunofluorescence (Middle) of LNCaP-SL cells in glial re-differentiation medium shows the acquisition of a glia-like morphology and increased expression of GFAP, a marker of glial cells. Conversely, LNCaP parental cells do not demonstrate increased GFAP expression after 14 days in re-differentiation medium (Right).

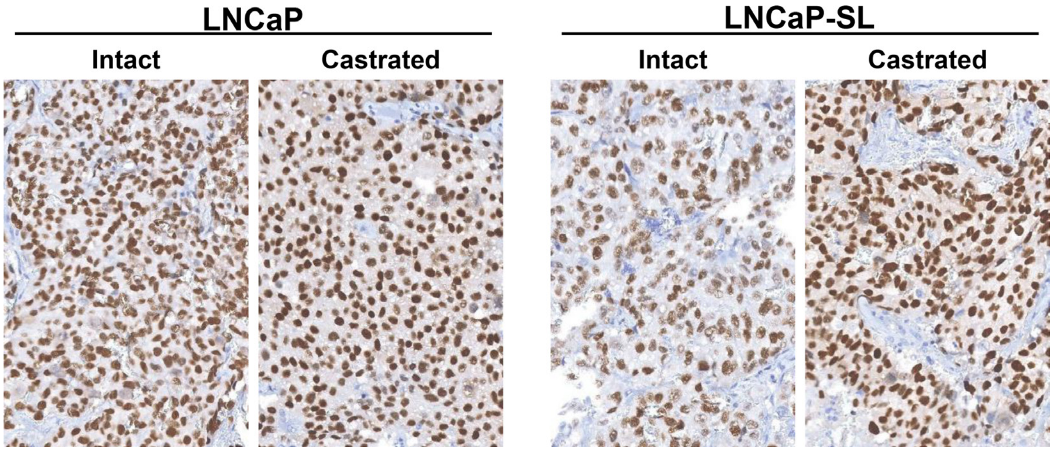

Supplementary Figure 4: Xenografted LNCaP-SL cells re-differentiate *in vivo* to AR^{+} cells. Immunostaining on sections of tumor xenografts confirms that tumours initiated by AR^{-} developmentally reprogrammed LNCaP cells in both intact and castrated mice form tumors that re-express AR.

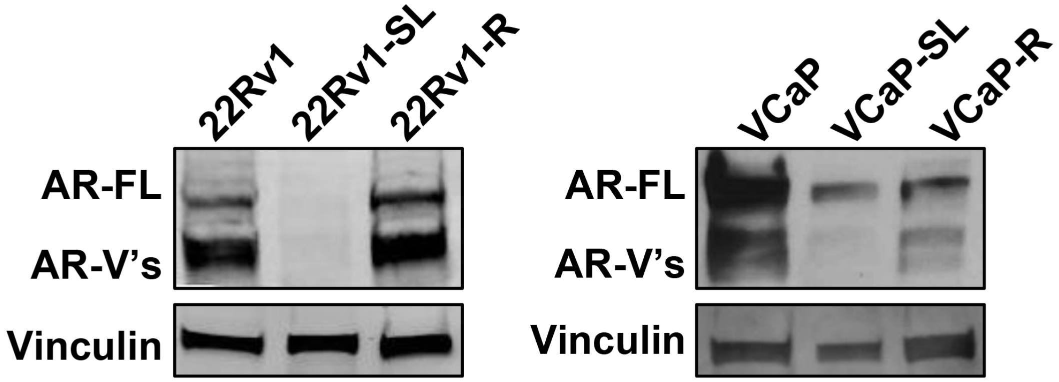

Supplementary Figure 5: Serum returned developmentally reprogrammed VCaP and 22Rv1 cells regain AR expression. Similar to LNCaP-SL, VCaP-SL and 22Rv1-SL cells cultured in serum re-express full-length and variant AR protein.

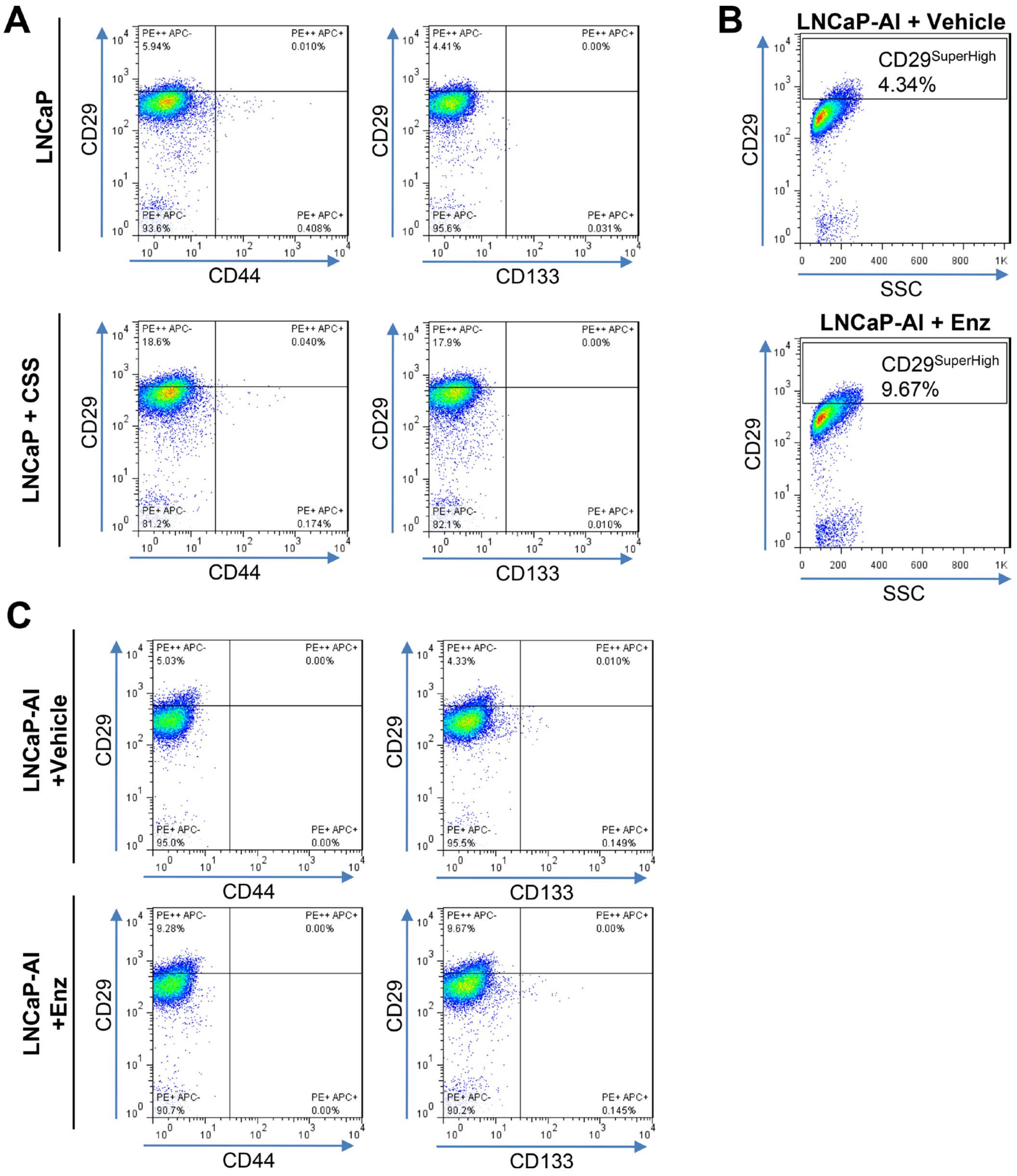

Supplementary Figure 6: Androgen deprivation does not enhance populations of putative CSC markers, CD133 or CD44. (**A–C**) While androgen deprivation significantly increases the population of $CD29^{SuperHigh}$ cells in LNCaP and LNCaP-AI cells, acute (72 hour) androgen-deprivation (CSS) in LNCaP cells, or 10μM enzalutamide-treatment in LNCaP-AI cells does not alter the relative populations of $CD44^{+}$ or $CD133^{+}$ cells.
