# Supplementary material for: Therapy-induced developmental reprogramming of prostate cancer cells and acquired therapy resistance
Source: Oncotarget. 2017 Jan 27;8(12):18949–67. doi: 10.18632/oncotarget.14850 (PMC5386661; doi:10.18632/oncotarget.14850)
Supplement: Supplementary file 2 [file oncotarget-08-18949-s002.docx]

**Supplementary Table S1: Annotated list of over-expressed Neural/Neural Crest (N/NC) related genes in LNCaP cells cultured 15 days in androgen deprived medium.**

| **Gene symbol** | **Annotation** | **Role** |
| --- | --- | --- |
| AMOTL1 | neural crest, cardiac development | N/NC Development |
| CCDC80 | migration of neural crest cells |  |
| CRIP2 | neural crest-derived cardiac development |  |
| EPAS1 | high in cns, regulates heart beat (Neural crest deriv) |  |
| EPB41L4A | neural crest, eye development |  |
| FBXL2 | neural crest, cochlear development |  |
| GAB3 | neuron / neural crest development |  |
| ID1 | marker of neural crest cells |  |
| ID3 | required for neural crest development |  |
| IGFBP3 | Involved in Neural Crest Cell migrations |  |
| LEF1 | development of neural crest lineages |  |
| NEDD9 | expressed in neural/neur crest precursors, filament org |  |
| SCD5 | expressed in neural crest and heart |  |
| SCUBE2 | neural crest, craniofacial development |  |
| SI | expressed in neural crest, gi derivatives |  |
| SPRY4 | Brain, Cranial Facial, Tooth Development |  |
| TSPAN12 | req for retinal development |  |
| CRISPLD1 | craniofacial development |  |
| COL5A2 | eye development |  |
| VIM | neurofilament org, high in cns, astrocyte form, eye, brain develop |  |
| ANKRD16 | assoc with brain complications causing tremors |  |
| BCOR | facial, cardiac, dental abnormalities |  |
| FRAS1 | craniofacial disorders, urogenital tract malformation |  |
| FZD4 | glioma, retinal disease (vascular), auditory sensing, stem cells |  |
| LEPREL1 | assoc with vitreoretinal degeneration disease |  |
| PRSS23 | cleft lip |  |
| RNF217 | dysfunction assoc with cleft palate, facial abnormal |  |
| ST3GAL5 | infantile epilepsy, developmental blindness |  |
| AGR2 | predisposition to brain infarctions |  |
| ATP8A1 | hippocampal development, brain function |  |
| ATXN1 | Notch repressor involved in neurogenesis |  |
| CALCRL | interacts with bmp, motor neuron development |  |
| CARTPT | promotes neuronal development and survival |  |
| CERK | neuronal differentiation and survival |  |
| CHN1 | primary expression in neuron |  |
| CNTNAP3 | cell surface interactions during cns development, neurite outgrowth |  |
| COBL | required for neural tube closure |  |
| DAB1 | cortical neuron development, neuronal migration |  |
| DAPK2 | isoform specific for stem cells |  |
| EFNA5 | nervous system development |  |
| GARNL3 | neuronal signaling, cortical development |  |
| GPR98 | CNS development & auditory function |  |
| ID2 | neural stem cells, differentiation and function |  |
| INTU | nervous system development |  |
| LGR4 | neural plate, neural tube closure, otic, optic nerve formation |  |
| NAB1 | repressor of EGR, Krok20 signaling, high in cns, required for brain development |  |
| NELL2 | growth and differentiation of neural stem cells |  |
| OFD1 | oral facial development reg ulator |  |
| PLXDC2 | neural stem cells, neuronal development |  |
| PTCH1 | Hedgehog Receptor |  |
| RTN4RL1 | axonal regeneration and plasticity |  |
| TRIM45 | high in embryonic brain |  |
| TSHZ3 | regulates respiratory neurons |  |
| ZBTB20 | required for hippocampal development |  |
| JAG1 | Notch ligand |  |
| NPTN | hippocampal synapses, synaptic plasticity |  |
| ABP1 | Dopamine Metabolism | N/NC Function |
| ADRA1A | alpha1 adrenergic receptor cns + smooth muscle contract |  |
| AK5 | highly expressed in cns; target in autoimmune limbic encephalitis |  |
| AKAP5 | high in cns/adrenal medulla, binds to beta-adrenergic receptor |  |
| AMIGO2 | survival of cerebellar granule neurons |  |
| AMY1A | high expression in pancreatic acinar cells |  |
| ARHGEF3 | high neuronal expression, neural |  |
| ATP11C | regulates ion transport, expressed in cns |  |
| BCAS1 | dopaminergic transmission / recover from parkinsons |  |
| BEX1 | regulates p75 NTR signaling |  |
| CA13 | expressed In neural tissues amongst others |  |
| CAMK1D | activates dendritic growth in hippocampal neurons |  |
| CAMK2N1 | synaptic protein |  |
| CAPS2 | synaptic vesicle release, hippocampal neurons |  |
| CCDC82 | hypothalmus, pituitary, adrenal |  |
| CLUL1 | retinal clusterin |  |
| CP | transferrin metabol, loss assoc with neuronal dysfunction |  |
| CRYM | neuronally expressed, defects lead to deafness |  |
| CTNNA3 | neural, cardiac functions |  |
| DAPK1 | modulates nmda receptors + neuronal apoptosis |  |
| DDC | dopamine, tryptamine synthesis |  |
| DMGDH | folate binding protein, craniofacial abnormalities |  |
| DPYD | mutations affect intellectual abilities |  |
| DST | anchors intermediate filaments in neurons |  |
| ENPP5 | neuronal cell-cell communication |  |
| ERV3 | expressed in adrenal cortex, CNS |  |
| FOSL2 | expressed in cns, modulates neuronal signaling |  |
| FSTL1 | bmp sig regulator, brain abnormal phenotypes |  |
| GAB1 | neurotrophin, insulin, erb signaling, exp in cns |  |
| GALNT10 | hypothalmus, thalmus, amygdyla |  |
| GLIPR1L2 | gli pathogenesis-like related, expressed in neuronal tiss |  |
| GPR63 | role in brain function |  |
| GPRC5B | GABA receptor-like, expressed in CNS |  |
| GRIK1 | glutamate receptor |  |
| HOXC9 | neuronal differentiation |  |
| HS6ST2 | heparin sulfate syn, neuronally expressed + high in brain |  |
| ICA1L | involved in amyotrophic lateral sclerosis |  |
| IL6ST | receptor complex for cntf, dopamine hydroxylase + cells |  |
| ITPR1 | gene linked to cerebellar disorders |  |
| KCNC2 | K+ gated channel, neuronal |  |
| KCNJ13 | K-channel associated with retinal dystrophy |  |
| KCNMB4 | Ca++ activated large K channel--GABA r positive cells |  |
| LRRC7 | organization of synaptic contacts |  |
| LRRN1 | neuronally expressed |  |
| MAP1B | microtubule formation, neurogenesis |  |
| MDGA1 | required for migration of cortical neurons/ nc cells |  |
| MET | neural migration, melanocytes, cardiac develop |  |
| MLL3 | paralogue of mll trithorax activator |  |
| MTSS1 | neural membranes, modulation of shh signaling |  |
| MTUS1 | assoc with ang2 receptor, expressed adrenal medulla, brain |  |
| MYOZ1 | calcineurin signaling, myogenesis, cardiomyopathy |  |
| MYRIP | involved in melanosome transport (melanocytes) |  |
| NCALD | rhodopsin phosphorylation |  |
| NFIL3 | neurofilament-maintenance of neuron |  |
| NIPSNAP3B | cns expression, vesicle trafficing |  |
| NLGN1 | synaptic junction formation, binds b-neurexins |  |
| OPRK1 | opoid receptor |  |
| OSBPL6 | lipid transport, expressed in cns |  |
| PCDH11X | synaptic junctions |  |
| PCDHB10 | synaptic maintenance |  |
| PCDHB13 | synaptic maintenance |  |
| PCDHB14 | synaptic maintenance |  |
| PGCP | enzyme involved in glutamate metabol |  |
| PIPOX | pipicolic acid oxidase deficiency--peroxisome disorder associated with loss of brain function |  |
| PLA2G2A | located in peripheral neural fibres |  |
| PRKG2 | expressed in amygdala, involved in anxiety behaviors |  |
| PTPRK | intercell adhesion, binds to neural adhesion molecules |  |
| PVRL3 | polio receptor, present at synapses, inter with matrix |  |
| RIMS1 | regulation of neurotransmitter release |  |
| RNF182 | ubiq-related targets synaptic vesicle protein |  |
| SAMD8 | sphingomyelin synthesis in neurons |  |
| SGK1 | regulates ion channels, highly expressed in neurons |  |
| SHC4 | migration of melanoma |  |
| SLC44A3 | choline transport in neurons |  |
| SNRK | neural control of dopamine signaling |  |
| SOS1 | MET receptor |  |
| STBD1 | cardiac, Parkinson's Disease |  |
| STON1-GTF2A1L | present at synaptic junctions-vesicle release |  |
| STON2 | synaptic vesicle formation and endocytosis (reuptake) |  |
| SYTL2 | neurotransmitter biosynthetic enzyme |  |
| TMEM38B | regulates intracell Ca++ stores, exp in CNS, cardiomyocytes |  |
| TMEM59L | brain specific, involved in APP processing |  |
| TSPAN7 | expressed in hippocampal neurons |  |
| TYRP1 | tyrosinase regulation (Melanoma) |  |
| USP25 | high in prolif neuroepithelial cells and post-mitotic neurons |  |
| VAMP4 | synaptic vesicle formation and dopamine release |  |
| VIPR2 | VIP receptor, neuronal expression throughout |  |
| TAS2R3 | taste receptor 2 BITTER | N/NC Lineages |
| TAS2R38 | taste receptor BITTER |  |
| TAS2R4 | taste receptor 2-4 BITTER |  |
| TAS2R44 | taste receptor-activated by saccharine (sweet-like) |  |
| VN1R1 | phermone receptor Olfactory-like sensory neuron |  |
| TRPC1 | neuronal, olfactory, cation channel regulates apoptosis |  |
| PTPLA | myopathies, cardiac dysfunction, exp in olfactory sensory neurons |  |
| PDE4DIP | develop of photoreceptor cells |  |
| OR13C2 | olfactory receptor 13c2 |  |
| OR13C5 | olfactory receptor |  |
| OR1F2P | olfactory receptor |  |
| MSRB3 | development of auditory sensations |  |
| ELOVL4 | photoreceptor FA synthesis gene, macular dystrophy |  |
| MCTP1 | Ca++ binding protein, taste receptor neurosensory cells |  |
| ODZ2 | neurestin alpha, teneurin (Odorant receptor cells) |  |
| APCDD1 | stem cell, involved in astro-gliogenesis |  |
| CD24 | axon guidance, defects in eye development other CNS dis |  |
| COLEC12 | reactive astrocytes, APP processing |  |
| CSMD1 | cleft/faciocranial malformations, neuritogenesis |  |
| ENO2 | neuron-specific enolase |  |
| LAMB1 | neurite outgrowth, axon formation |  |
| NR5A2 | steroidogenesis, pancreas development |  |
| NRG3 | ligand for erbb4, oligodendrocyte survival |  |
| NTN4 | promotes neurite extension from olfactory bulb |  |
| NTNG1 | Promotes Neurite Outgrowth |  |
| PEBP4 | involved in myoblast differentiation |  |
| PLCH1 | signaling in neuronal/neuroendocrine cells |  |
| PNMA2 | onconeuronal antigen, small intestine neuroendocrine |  |
| QKI | req for myelinization; defects in myelin sheath and oligodend function |  |
| ROR2 | chondrocyte lineage and subsequent bone formation |  |
| SEMA6A | axonal guidance |  |
| SERPINI1 | neuroserpin, formation of synaptic connections |  |
| SLIT1 | axonal navigation |  |
| SLITRK3 | Slit and Ntrk-like protein regulates neurite outgrowth |  |
| ST6GALNAC5 | ganglioside maturation, neuronal, brca mets to brain |  |
| SUSD4 | oligodendrocytes, axons, eye development, brain |  |
| ZFHX4 | transcription, neural and muscle differentiation |  |
