# Supplementary material for: Therapy-induced developmental reprogramming of prostate cancer cells and acquired therapy resistance
Source: Oncotarget. 2017 Jan 27;8(12):18949–67. doi: 10.18632/oncotarget.14850 (PMC5386661; doi:10.18632/oncotarget.14850)
Supplement: Supplementary file 3 [file oncotarget-08-18949-s003.docx]

**Supplementary Table S2: List of the developmentally reprogrammed PCa cell line derived 132-gene signature.**

| **Gene Symbol** | **Probe Name** |
| --- | --- |
| MMP10 | A_23_P13094 |
| THSD7A | A_24_P400324 |
| EGR1 | A_23_P214080 |
| ETV5 | A_32_P30649 |
| RNLS | A_23_P202501 |
| DSC1 | A_23_P38696 |
| IFI27L2 | A_23_P140146 |
| PLOD2 | A_33_P3318581 |
| LAMB3 | A_33_P3338121 |
| FOXN4 | A_32_P140030 |
| CHST2 | A_23_P40847 |
| MGP | A_33_P3361636 |
| TYRP1 | A_23_P94403 |
| MRC2 | A_33_P3364741 |
| SYTL2 | A_23_P53193 |
| TNFRSF11B | A_23_P71530 |
| MET | A_23_P359245 |
| ITGB8 | A_24_P759477 |
| RCAN2 | A_32_P156851 |
| SPTLC3 | A_24_P49260 |
| CD86 | A_24_P131589 |
| PDE4DIP | A_33_P3333982 |
| NTN4 | A_23_P204630 |
| NAB1 | A_24_P191417 |
| OPRK1 | A_32_P33576 |
| SULF1 | A_23_P43164 |
| A2M | A_23_P116898 |
| ANKRD29 | A_23_P412577 |
| DMKN | A_23_P320261 |
| C2orf54 | A_23_P60990 |
| SHC4 | A_32_P234145 |
| LAMA3 | A_33_P3262560 |
| BCHE | A_23_P212050 |
| MMP1 | A_23_P1691 |
| PDE4DIP | A_33_P3246985 |
| MYB | A_23_P31073 |
| SPTLC3 | A_33_P3415395 |
| PCDHB9 | A_23_P18798 |
| SIPA1L2 | A_23_P137470 |
| HMBOX1 | A_24_P932736 |
| DOCK10 | A_23_P16722 |
| ADRB2 | A_23_P145024 |
| NAV2 | A_23_P52727 |
| EMP1 | A_23_P76488 |
| AMIGO2 | A_23_P14083 |
| PHLDA2 | A_23_P47614 |
| PCDHB10 | A_23_P7397 |
| CA13 | A_23_P381714 |
| ZYG11A | A_32_P132589 |
| TYRP1 | A_33_P3399980 |
| HTRA1 | A_23_P97990 |
| SLC9A9 | A_32_P46214 |
| CLIC5 | A_23_P416774 |
| NOVA1 | A_33_P3347697 |
| AMTN | A_33_P3212860 |
| ROR2 | A_23_P158318 |
| SYCE2 | A_32_P203528 |
| NNMT | A_23_P127584 |
| LEPREL1 | A_23_P69179 |
| ENTPD8 | A_33_P3420224 |
| MRAP2 | A_23_P357207 |
| AGT | A_23_P115261 |
| SPG20 | A_33_P3352877 |
| LHFPL2 | A_23_P255104 |
| C16orf45 | A_23_P326319 |
| FAM65B | A_24_P941359 |
| LUM | A_23_P99063 |
| TSPAN8 | A_23_P36531 |
| ADRA2A | A_23_P138706 |
| GSN | A_33_P3423365 |
| LAMA3 | A_23_P89780 |
| NOVA1 | A_24_P270496 |
| SEPW1 | A_23_P27724 |
| UST | A_23_P314145 |
| SOX9 | A_23_P26847 |
| ATP1B1 | A_23_P62932 |
| MYOF | A_23_P354387 |
| KCNN4 | A_23_P67529 |
| KRT17 | A_23_P96158 |
| CA12 | A_24_P330518 |
| EGLN3 | A_33_P3256952 |
| FGF12 | A_24_P334300 |
| SEPW1 | A_33_P3326432 |
| KCNK13 | A_23_P3177 |
| SATB1 | A_23_P259741 |
| MAP1B | A_24_P879740 |
| HIP1R | A_33_P3269924 |
| CAPS2 | A_32_P209208 |
| AUTS2 | A_33_P3330209 |
| KIAA1377 | A_32_P58614 |
| C8orf47 | A_23_P333029 |
| CRYM | A_23_P77731 |
| MSMB | A_24_P146683 |
| UNC5B | A_23_P52336 |
| PLSCR1 | A_23_P69109 |
| SGK1 | A_23_P19673 |
| ASGR1 | A_23_P118722 |
| PPAPDC1A | A_24_P810290 |
| SLPI | A_24_P190472 |
| GALNT7 | A_23_P144384 |
| NR5A2 | A_33_P3389728 |
| OR51E1 | A_33_P3424491 |
| ARSJ | A_33_P3296582 |
| ACVR1C | A_23_P397455 |
| SORD | A_33_P3413483 |
| LEFTY1 | A_23_P160336 |
| SLC16A3 | A_23_P158725 |
| KRT16P2 | A_32_P62963 |
| OR51E1 | A_23_P13493 |
| CDH26 | A_23_P502957 |
| DSEL | A_24_P365180 |
| TRIM2 | A_24_P208909 |
| RGS5 | A_33_P3243093 |
| S100A14 | A_23_P124619 |
| IFITM1 | A_23_P72737 |
| TFRC | A_23_P212617 |
| ITGA1 | A_33_P3353791 |
| IL15 | A_23_P29953 |
| CPAMD8 | A_23_P67198 |
| SLC22A18 | A_23_P139260 |
| OR51E2 | A_24_P235756 |
| SPINK1 | A_23_P214079 |
| PRKCD | A_23_P144054 |
| FBXL7 | A_23_P144827 |
| FMO5 | A_23_P233 |
| MATN2 | A_23_P71328 |
| RIMS1 | A_33_P3218832 |
| ELL2 | A_23_P58506 |
| RHOH | A_23_P58132 |
| IGF2 | A_23_P150609 |
| UGT1A6 | A_23_P60599 |
| ARHGAP10 | A_23_P21548 |
| QSOX1 | A_23_P12463 |
| PSMB8 | A_23_P250629 |
| CACNA1D | A_23_P365767 |
| ALCAM | A_33_P3713357 |
| KCTD12 | A_33_P3240512 |
| KIAA1462 | A_23_P301521 |
| DBP | A_23_P130753 |
